# Central Mongolian lake sediments reveal new insights on climate change and equestrian empires in the Eastern Steppes

**DOI:** 10.1038/s41598-022-06659-w

**Published:** 2022-02-18

**Authors:** Julian Struck, Marcel Bliedtner, Paul Strobel, William Taylor, Sophie Biskop, Birgit Plessen, Björn Klaes, Lucas Bittner, Bayarsaikhan Jamsranjav, Gary Salazar, Sönke Szidat, Alexander Brenning, Enkhtuya Bazarradnaa, Bruno Glaser, Michael Zech, Roland Zech

**Affiliations:** 1 Department of Geography, Physical Geography, Friedrich Schiller University Jena, Jena, Germany; 2 University of Colorado-Boulder Museum of Natural History, Boulder, CO 80309 USA; 3 Department of Geography, Geographic Information Science, Friedrich Schiller University Jena, Jena, Germany; 4 Section Climate Dynamics and Landscape Evolution, GFZ German Research Centre for Geosciences, Potsdam, Germany; 5 Department of Geology, University of Trier, Trier, Germany; 6 Department of Soil Science, University of Trier, Trier, Germany; 7 Institute of Geography/ Physical Geography with Focus on Paleoenvironmental Research, Technische Universität Dresden, Dresden, Germany; 8 Institute of Agricultural and Nutritional Sciences, Soil Biogeochemistry, Martin Luther University Halle-Wittenberg, Halle (Saale), Germany; 9 Department of Archaeology, Max Planck Institute for the Science of Human History, Jena, Germany; 10 Department of Chemistry, Biochemistry and Pharmaceutical Sciences and Oeschger Centre for Climate Change Research, University of Bern, Bern, Switzerland; 11 Institute of Plant and Agricultural Sciences, School of Agroecology and Business, Mongolian University of Life Sciences, Darkhan, Mongolia

**Keywords:** Climate-change impacts, Environmental impact, Socioeconomic scenarios

## Abstract

The repeated expansion of East Asian steppe cultures was a key driver of Eurasian history, forging new social, economic, and biological links across the continent. Climate has been suggested as important driver of these poorly understood cultural expansions, but paleoclimate records from the Mongolian Plateau often suffer from poor age control or ambiguous proxy interpretation. Here, we use a combination of geochemical analyses and comprehensive radiocarbon dating to establish the first robust and detailed record of paleohydrological conditions for Lake Telmen, Mongolia, covering the past ~ 4000 years. Our record shows that humid conditions coincided with solar minima, and hydrological modeling confirms the high sensitivity of the lake to paleoclimate changes. Careful comparisons with archaeological and historical records suggest that in the vast semi-arid grasslands of eastern Eurasia, solar minima led to reduced temperatures, less evaporation, and high biomass production, expanding the power base for pastoral economies and horse cavalry. Our findings suggest a crucial link between temperature dynamics in the Eastern Steppe and key social developments, such as the emergence of pastoral empires, and fuel concerns that global warming enhances water scarcity in the semi-arid regions of interior Eurasia.

## Introduction

The rise of transcontinental, pastoral empires linking eastern and western Eurasia across the steppes had a tremendous transformative effect on human societies, facilitating the spread of people, goods, and ideas — as well as organisms like domestic animals, plants, and catastrophic disease^1–4^. The Mongolian steppe was first occupied by pastoral people ca. 3000 BCE, when early herders appear to have migrated to the region from western Asia^5–7^. Around 1200 BCE, domestic horses were used first for transport by mobile herders of the Deer Stone–Khirgsuur complex (DSK) and other Bronze Age culture groups^8–11^. The emergence of horse culture changed mobility of the steppe cultures, leading to the rise of important nomadic polities like the Xiongnu (ca. 200 BCE–100 CE) and the Great Mongol Empire, who rose to global dominance under Genghis Khan in the early thirteenth century CE^10,12^. For these pastoral empires, extensive and productive grasslands provide the engine for both economic and political power^13,14^. Yet, particularly in the dry and harsh steppes of eastern Eurasia, minor climate variations can have large impacts on the water balance, biomass production, and ecosystem carrying capacity^15–17^. The close coupling between precipitation and temperature regimes and domestic animal productivity has inspired hypotheses that climate changes may have played an important role for network formation and human history in Central Asia^17^. While social-economic changes such as the emergence of social inequality in pastoral societies can be well inferred from historical and archaeological records^11,17,18^, potential climatic controls can only be assessed by high-resolution paleoclimate records, which are currently rare for the Late Holocene in Mongolia.

Paleoclimate information derived largely from lake sediments^19–26^ and tree-rings^13,27–29^, suggest a possible link between the onset of wetter conditions and social integration among Mongolian pastoralists. For northern and central Mongolia, many records indicate a shift to humid conditions with the onset of the Late Holocene. Bliedtner et al.^30^ linked the dispersal of mobile pastoralists in the Altai Mountains to warm and humid conditions between 850 and 1550 BCE. Increasing moisture availability around 1000 BCE^19,31^ was suggested to favor the expansion of nomadic tribes^32^. For the past ~ 1500 years high resolution tree-ring records show short-term temperature fluctuations^27–29,33^, that can be attributed to volcanic forcing^34,35^. Di Cosmo et al.^36^ identified favorable humid conditions at the emergence of the Uyghur Empire followed by a persistent drought between 783 and 850 CE. Pederson et al.^13^ identified more persistent droughts during the Medieval Climate Anomaly (MCA; 850–1300 CE), followed by warmer and more humid conditions between 1211 and 1225 CE, which could have favored the expansion of the Mongol Empire.

Despite these tantalizing links between climate changes and pastoral dynamics, the paleoclimatic and -environmental records for Mongolia suffer from poor temporal resolution, age uncertainties, and/or ambiguous proxy interpretation. Oftentimes, chronological frameworks designed for geological research questions (with wide error ranges) are applied to archaeological timescales, meaning that the same dataset can be used to draw widely differing conclusions^37^. Anthropogenic impacts related to herding have also drastically impacted Mongolia’s landscape — meaning that pollen and any other biological data, for example, might be affected by human land-use since 1200 or even 3000 BCE^8,31^, which could hamper a direct paleohydrological reconstruction. In order to provide a more convincing link between climate and human history, more robust and well-dated high-resolution paleoclimate records are needed. Here, we report a single well-dated paleohydrological record that spans the whole timeline of Mongolian pastoral history and prehistory from the late Bronze Age, allowing the first proper opportunity to test for a causal link between climate dynamics and pastoral empires in the eastern Steppe.

**Figure 1:**
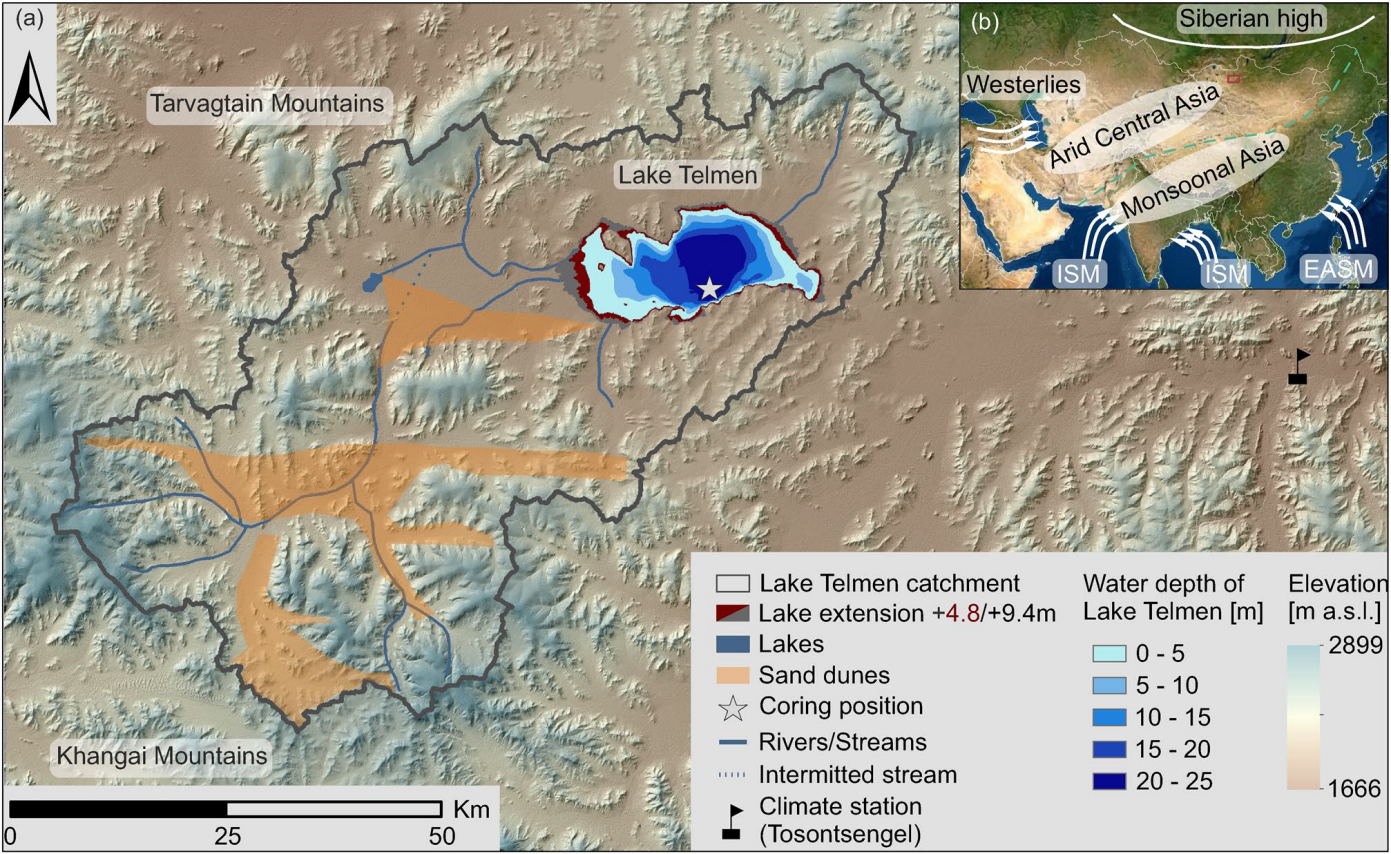
Regional setting of Lake Telmen. (**a**) Digital elevation (SRTM, 1 arc second; 30 m × 30 m) and bathymetric data show the catchment’s topography and the hydrography of Lake Telmen. The red/grey-shaded area around Lake Telmen indicates the former lake extent during the + 4.8 m/+ 9.4 m lake level high-stands (Peck et al.^22^), the yellow-shaded areas indicate the extent of sand dunes. The coring position is marked by a grey star, and the climate station Tosontsengel by a black flag. (**b**) Inset map of Asia shows the extent of “Arid Central Asia” and “Monsoonal Asia”, the northern Monsoon boundary is illustrated by a green dashed line. The Mongolian study site is marked by a red box. The direction of major atmospheric circulation systems is illustrated by white arrows: The Westerlies, the East Asian summer monsoon (EASM), the Indian summer monsoon (ISM) and the Siberian high. The maps were created using ArcMap 10.6.1 (www.esri.com; (**a**)) and SimpleMappr (www.simplemappr.net; (**b**)). In-map labels (mountains, atmospheric circulation systems) were added in CorelDraw 2019 (www.coreldraw.com).

**Figure 2:**
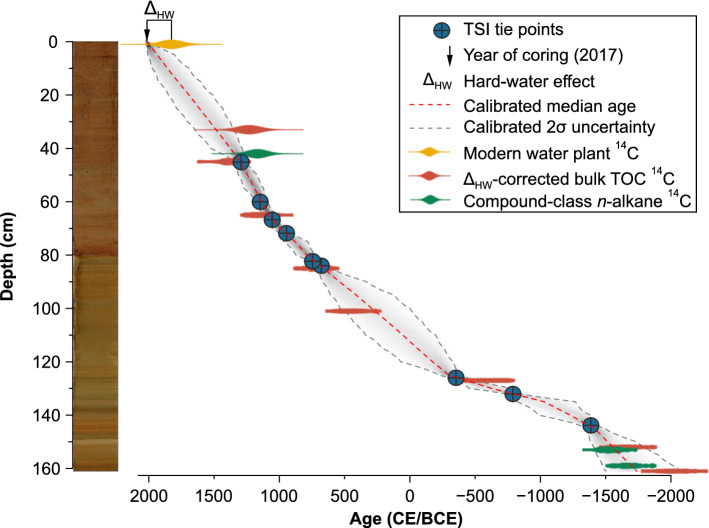
Photo of the sediment core from Lake Telmen, taken after oxidation, and age-depth model. The age-depth model is based on tie-points (blue dots) with the total solar irradiance (TSI)^44^ (Supplementary Fig. 2). The ^14^C ages are plotted for comparison. The modern hard-water effect (Δ_HW_) is the difference between the year of coring in 2017 and the ^14^C age of a modern water plant (yellow). Δ_HW_-corrected bulk TOC ^14^C ages and compound-class *n*-alkane ^14^C ages are shown in red and green, respectively (Supplementary Table 1).

In this study, we investigated a 161 cm long sediment core from Lake Telmen in semi-arid central Mongolia (Figs. 1, 2). We applied radiocarbon dating on bulk TOC and molecular markers to establish a robust chronology, and we combined compound-specific δ^2^H and δ^13^C on individual *n*-alkanes, bulk δ^13^C and δ^18^O on carbonates, with elemental and inorganic geochemical and sedimentological analyses to establish a detailed paleoenvironmental record and to precisely constrain the regional hydrological history. Moreover, the lake’s sensitivity to changes in temperature and precipitation was evaluated by a hydrological water balance model, which enables an identification of relevant forcings.

## Regional setting and site description

Lake Telmen Nuur (Nuur = Lake) is a hyposaline oligotrophic, closed-basin lake in Zavkhan province in semi-arid central Mongolia, along the western edge of the Khangai Mountains (Fig. 1a). The lake is fed by precipitation and small streams, which are draining the Khangai and Tarvagtain Mountains. The modern lake has an area of 209 km^2^ and a maximum water depth of 25 m. The lake catchment has an area of 3761 km^2^ and covers altitudes from 1788 to 2755 m a.s.l. (Fig. 1a). The catchment geology corresponds to the Permian–Triassic volcanic-plutonic belt and its vegetation belongs to the steppe and forest-steppe biome^31^, characterized by *Poaceae*, *Cyperaceae*, *Artemisia* spp. and *Caragana* spp. Only the barchan dunes in the western part are covered by patches of *Larix sibirica*^24,38^. Today, mean annual precipitation (MAP) varies from 150 to 444 mm a^−1^ (Tosontsengel climate station) and maximum precipitation occurs in June, July, and August (1990–2020). Mean monthly precipitation is 3 mm in January and 63 mm in July. Mean annual temperature (MAT) varies from − 6.8 to − 3.6 °C and coldest temperatures of − 32 °C on average occur in January, while warmest average temperatures of 16 °C were measured in July (1990–2020). Compared to previous periods (1961–1990), the summer climate became drier and warmer during the last decades, and the potential evapotranspiration of ~ 760 mm a^−1^ distinctly exceeded MAP^39^. Climate is mainly controlled by its continental position at the intersect of three atmospheric circulation systems: the mid-latitude Westerlies, the East Asian summer monsoon (EASM), and the Siberian high, which affect the amount of precipitation, moisture availability^40,41^, and the cold and dry winter climate^42^. Thus, Mongolia is a transition area between Westerly-dominated “Arid Central Asia” and “Monsoonal Asia” (Fig. 1b)^40^. Today, the summer precipitation regime is mainly controlled by the Westerlies and small-scale spatial convection bringing recycled moisture from the EASM-dominated region^40^. The influence of the EASM on Mongolia is controversially discussed^31,40^, and may have been more important during the MCA^43^ and periodically during the late Holocene (since 2000 BCE)^15,40^ (Supplementary Sect. S7). The harsh winter climate is dominated by the Siberian high^42^, which enables an ice cover of five to six months per year (Planet Team: 2016–2019).

## Results

### Sediment core chronology

The lowermost and oldest radiocarbon age from our sediment core is 2300 ± 170 BCE, while a present-day water plant reveals a hard-water effect (Δ_HW_) of 190 ± 83 years (Supplementary Table 1). We established an age-depth model (ADM) using seven Δ_HW_-corrected bulk ^14^C ages and two compound-class *n*-alkane ^14^C ages (Supplementary Fig. 1). The ^14^C chronology is stratigraphically very consistent (more detailed information is provided in Supplementary Sect. S1). We further refined the ADM using nine tie points that we identified by comparison with total solar irradiance (TSI)^44^ (Fig. 2, Supplementary Fig. 2, see “Methods” section). The (Δ_HW_-corrected) ^14^C ages overlap with the 95% confidence interval of the tie-point ADM, and the median ages of both ADMs differ by no more than 246 years.

### Sedimentological and geochemical analyses

**Figure 3:**
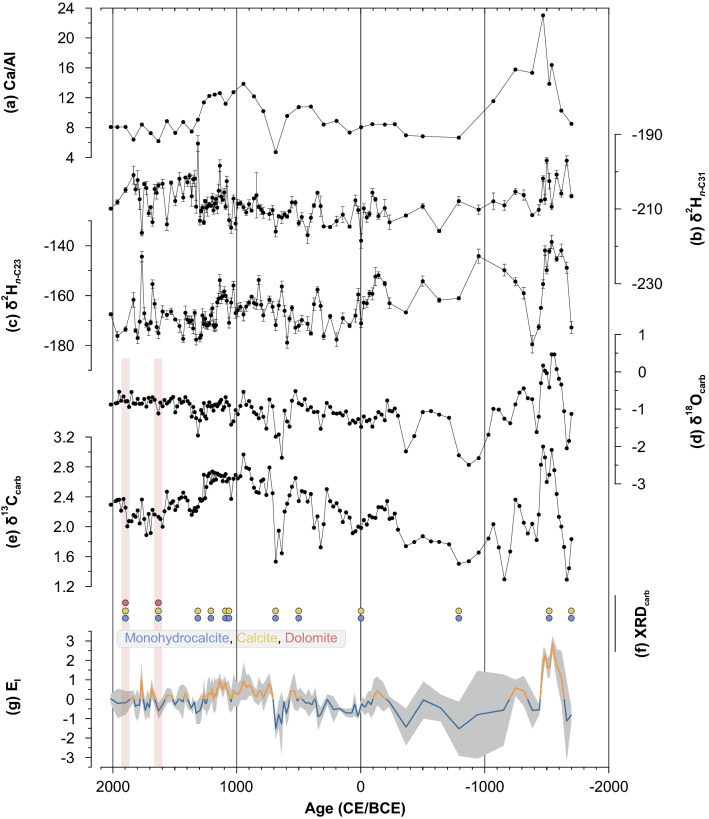
Geochemical and isotope records for the sediment core from Lake Telmen. (**a**) The Ca/Al ratio [–], (**b**) compound-specific δ^2^H_*n*-C31_ [‰ vs. VSMOW], (**c**) compound-specific δ^2^H_*n*-C23_ [‰ vs. VSMOW], (**d**) bulk carbonate δ^18^O [‰ vs. VPDB], (**e**) bulk carbonate δ^13^C [‰ vs. VPDB], (**f**) carbonate mineral forms; monohydrocalcite (blue), calcite (yellow), and dolomite (red). Red bars emphasize the occurrence of dolomite and a potential influence on bulk carbonate δ^18^O and δ^13^C, and (**g**) evaporation index (E_I_) [–], yellow = dry, blue = humid, gray-shaded area = standard deviation.

Our sediment core is finely laminated, mainly consists of silty siliciclastic components (≥ 66%) and is characterized by high amounts of total organic carbon (TOC: 5.4–12.3%) and carbonates (total inorganic carbon, TIC: 3.2–6.3%) (Supplementary Fig. 3). The carbonates are dominated by monohydrocalcite (MHC) and calcite (Fig. 3f). Minor contributions of dolomite are identified in the upper part of the sediment core (Fig. 3f), while gypsum could not be identified within the analyzed samples (more detailed information is provided in Supplementary Sect. S2, Supplementary Figs. 3, 5). Element analyses show significant correlations (α = 0.05) for Al, Fe, and K, for Mg and Na, and for Ca and Sr, respectively (Supplementary Table 2). The first principal component (PC1) describes 53.3% of the variance and shows strong positive loadings for Al, Fe, and K. PC2 describes 36.1% of the variance and has strong positive loadings for Na and Mg, and strong negative loadings for Ca (Supplementary Fig. 4). PC1 can be interpreted to reflect allochthonous input related to weathering and erosion processes in the catchment^45^, whereas PC2 characterizes predominantly the autochthonous precipitation of salts and carbonates (Supplementary Fig. 4). With regard to our geochemical data, the most relevant paleohydrological information is inferred from the Ca/Al ratio, which ranges from 4.7 to 23. Wide ratios indicate enhanced autochthonous production and carbonate precipitation during probably dry and warm periods around 1500 BCE and again around 1000 CE (Fig. 3a). Narrow ratios indicate more allochthonous input in between 1200 BCE and 700 CE, as well as after ~ 1300 CE, which can be interpreted to document humid conditions with elevated runoff.

### Isotope analyses, evaporation index (E_I_), and paleohydrology

*n*-Alkanes were present in all samples in sufficient amounts for compound-specific isotope analyses (Supplementary Fig. 6). A differentiation of allochthonous and autochthonous compounds can be made on the basis of *n*-alkane chain lengths. *n*-C_31_-Alkanes are predominantly synthesized by higher terrestrial plants and are of allochthonous origin^38,45^. A recently performed calibration study found that δ^2^H_*n*-C31_ reflects the isotopic signature of precipitation and shows no distinct amount- or temperature effect in Mongolia^46^. Therefore, δ^2^H_*n*-C31_ indicates different moisture sources (Westerlies vs. EASM) at Lake Telmen. δ^2^H_*n*-C31_ values range from − 219 ± 2.0 to − 193 ± 2.1‰ (Fig. 3b, more detailed information and discussion regarding the influence of different moisture sources are provided in Supplementary Sects. S3 and S7). *n*-C_23_-Alkanes, on the other hand, are predominantly synthesized by aquatic plants^45^. Thus, our δ^2^H_*n*-C23_ record reflects the isotopic signature of lake water and its evaporative ^2^H enrichment^47–50^. δ^2^H_*n*-C23_ ranges widely from − 180 ± 3.5 to − 139 ± 2.7‰ and indicates lake water ^2^H enrichment in distinct periods, particularly before 1500 BCE (Fig. 3c).

δ^18^O_carb_ ranges from − 2.5 to 0.5‰, reflecting also the isotopic signature and evaporative ^18^O enrichment of lake water^51–53^. Since the carbonates in Telmen Nuur consists of different mineral phases — monohydrocalcite, calcite, dolomite — an isotope enrichment due to higher contributions of dolomite (~ + 3‰)^54^ is likely, which however, was not observed in our record (Fig. 3f). Like δ^2^H_*n*-C23_ the δ^18^O_carb_ record shows maximum enrichment before 1500 BCE (Fig. 3d). Significant co-variation between δ^18^O_carb_ and δ^13^C_carb_ (r = 0.61, *p* = 1.06e^−17^) reflects evaporation under equilibrium conditions of dissolved and atmospheric CO_2_ and indicates the paleohydrological sensitivity of both isotopes^51–53^. δ^13^C_carb_ ranges from 1.3 to 3.1‰ with maximum values again before 1500 BCE, but also relatively high values around 1000 CE (Fig. 3e).

δ^2^H_*n*-C23_, δ^18^O_carb_, and δ^13^C_carb_ show similar down-core trends and primarily reflect the evaporative enrichment of lake water. We therefore combined all three proxies into a normalized Evaporation Index (E_I_; Fig. 3g, Supplementary Fig. 7, more detailed information is provided in Supplementary Sect. S5). Positive E_I_ values document enhanced evaporative lake water enrichment under dry conditions, which also leads to reduced moisture effectively available for the ecosystem at our study site. We refer to this as “dry” in the following. By contrast, negative E_I_ values document reduced evaporative enrichment due to colder and/or more humid conditions, which leads to increased effective moisture in this ecosystem (referred to as “humid”).

As illustrated in Fig. 3g, the E_I_ reveals dry conditions for the early Late Holocene until ca. 1200 BCE, followed by a long-lasting humid period from 1200 BCE to 700 CE. From 700 to 1300 CE, regional climate conditions became drier again, temporally coinciding with the MCA. With the onset of the Little Ice Age (LIA) around 1300 CE, regional climate tended to be more humid. This dry–humid–dry–humid pattern perfectly agrees with the Ca/Al ratio, corroborating the interpretation and robustness of the various proxies.

**Figure 4:**
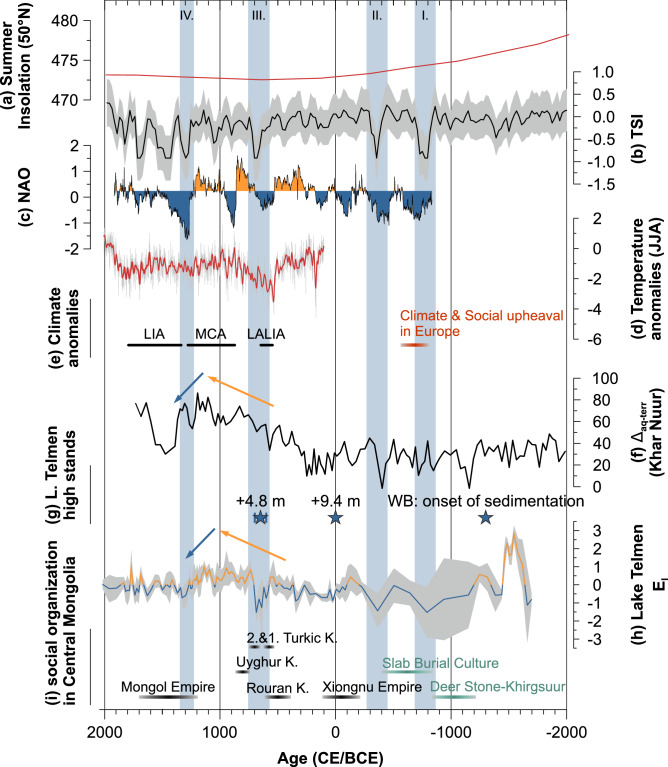
Late Holocene hydrological changes at Lake Telmen. (**a**) 50° N summer insolation [Wm^−2^]^88^, (**b**) total solar irradiance [Wm^−2^], gray-shaded area = 1σ uncertainty^44^, (**c**) North Atlantic Oscillation (NAO) index^58^ [–], (**d**) reconstructed June, July, and August temperature anomalies from the Russian Altai (°C, with reference to 1961–1990)^33^. Bold red line shows a 21 year moving average, (**e**) climate anomalies during the Late Holocene: Late Antique Little Ice Age (LALIA), Medieval Climate Anomaly (MCA), and Little Ice Age (LIA) after Büntgen et al.^33^ and the period of social upheaval in Europe^68,71^, (**f**) Δ_aq-terr_ — the offset between δ^2^H_*n*-C23_ (aquatic) and δ^2^H_*n*-C31_ (terrestrial) indicating lake water evaporative enrichment^30^ [‰ vs. VSMOW], (**g**) blue stars show lake level high-stands at Lake Telmen of 4.8 m and 9.4 m above the current lake level and the onset of sedimentation in the western basin (WB)^22^, (**h**) evaporation index (E_I_) [–], gray-shaded area = standard deviation, (**i**) social organization and duration of important Mongolian steppe cultures: Deer Stone–Khirgsuur complex (DSK), Slab Burial Culture^72^, the Xiongnu^10,75^, the Rouran Khaganate^10^, the first and second Turcik Khanganate^10,76^, the Uyghur Khanganate^36^ and the Mongol Empire^10,13^. Vertical blue bars indicate distinct solar minima at ~ 800 BCE (I.), 400 BCE (II.), as well as ~ 700 CE (III.) and ~ 1300 CE (IV.).

Our results show a transition from dry to humid conditions around 1200 BCE, associated with directional socio-economic changes in Mongolian pastoralist cultures^8,9^. Wetter conditions are associated with the onset of sedimentation in the western shallow lake basin previously dated to 1300 BCE^22^ and a prominent lake level rise of 9.4 m indirectly dated to ca. 0 CE (Figs. 1, 4g)^22^. Another lake high-stand is recorded by a prominent + 4.8 m shoreline terrace, which has been directly dated to between 600 and 700 CE^22^ and very likely points to a humid phase shortly before the onset of the drier MCA (Figs. 1, 4g).

## Discussion

### External forcing on the regional climate

Our results suggest that these long-term humidity trends are driven by changes in solar insolation (Fig. 4a). On the one hand, reduced summer insolation leads to a weakening of the monsoon system and thus to less precipitation in the areas affected by the monsoonal system and higher precipitation in the adjacent northern regions (sometimes referred to “Arid Central Asia”) and the transition area comprising Mongolia^40^ (Fig. 1). This effect can be explained with weak subsidence and more prominent convection. On the other hand, a long-term reduced summer insolation (~ 6 Wm^−2^ from the Mid- to the Late Holocene, Fig. 4a) will inevitably lead to lower temperatures, reduced evaporation and more effective ecosystem moisture (Supplementary Sect. S6).

However, superimposed on this long-term trend we find a strong resemblance of our E_I_ record with short-term fluctuations of the high-resolution TSI record^44^ (Fig. 4b,h). The solar minima at ~ 800 BCE, 400 BCE, as well as ~ 700 CE and after 1300 CE all coincide with prominent minima displayed by our E_I_ record. We interpret this as strong empirical evidence for solar forcing being the most important factor influencing the local hydrological conditions at our site.

TSI varies by about 1 Wm^−2^, which modifies the radiative forcing at the Earth’s surface and the mean global temperature by ~ 0.17 Wm^−2^ and ~ 0.07 °C, respectively^55^. Lean^56^ suggested a higher sensitivity for the mid-latitudes and regional temperature modifications of up to 2 °C. A temperature decrease at this magnitude should entail a significant decrease of evaporation, which directly affects the regional moisture balance (see “Hydrological modeling” section). In addition, reduced TSI during solar minima favored northern hemispheric cooling, which strengthened latitudinal temperature gradients and the Westerly jet streams^55,57,58^. Then, the North Atlantic Oscillation (NAO) tends to be in a distinct negative mode (Fig. 4c), which forces the Westerly jets to the South, advecting more moisture to “Arid Central Asia”, and thus, our research site^21,58^ (Fig. 1). In contrast, higher TSI leads to reduced latitudinal temperature gradients, which coincides with a distinct positive NAO mode (Fig. 4b,c) as well as a northward shift of the Westerly jets^21,58^, which favors dry conditions at Lake Telmen.

### Hydrological modeling

To evaluate how temperature and precipitation changes related to solar forcing could have impacted the water balance of Lake Telmen, we set up a hydrological model (Supplementary Table 3). The model-simulated long-term average water balance of Lake Telmen indicates a state close to equilibrium, which coincides well with the relatively stable average lake extent of Lake Telmen over the past decades^59^. Several sensitivity tests show that the water balance is highly sensitive to changes in temperature and precipitation (Supplementary Table 4). A 1 °C decrease in air temperature (and lake surface temperature, respectively), for example, reduces lake evaporation by already 5% or 8%, respectively.

During the lake high-stand of + 4.8 m above present (600–700 CE)^22^, the lake area was 13% larger than today (Figs. 1, 4g). To maintain the corresponding water balance state close to equilibrium, air temperature would need to be 0.35 °C lower, or precipitation would need to be 2.5% higher (~ 12 mm a^−1^) compared to present-day conditions. Considering the joint effect of air and water temperatures, a decrease of only 0.15 °C would already be sufficient. This highlights that even small temperature changes related to solar forcing (Fig. 4b) could explain the observed hydrological changes and lake level fluctuations. The + 4.8 m lake high-stand could therefore be explained by the solar minimum at ~ 700 CE (Fig. 4b,g), while age uncertainties in this part of the core section do not rule out a potential correlation of this particular lake high-stand with the Late Antique Little Ice Age (LALIA; 536–660 CE)^33^, which marks the onset of prominent temperature anomalies within the common era^60^. Major volcanic eruptions may have reduced temperatures by up to 2.5 °C at 536 CE and 543 CE^34,35^. Our hydrological model suggests that such a strong, short-term temperature decline would maintain a water-balance state close to equilibrium — even if precipitation decreased by 18% relative to present-day conditions. All this confirms that Lake Telmen and the hydrological water balance at the study site are extremely sensitive to solar forcing and even small temperature changes.

### Climate impact on human history in Mongolia

Sustained humid conditions likely enabled the expansion of fertile grasslands and thus, increased ecosystem carrying capacity^14,17,61^ — allowing to raise larger numbers of livestock and horses for both meat and dairy production^9,11^. Particularly in the dry and seasonal steppe environment, domestic livestock herds experience “economies of scale” — wherein smaller herds are more vulnerable to loss from disease, predation, or weather, and larger herds are more resilient^62^. With productive areas distributed unequally across the landscape, and some herders inevitably subject to disaster and loss, periods of environmental productivity appear to encourage the formation of larger steppe social networks^17^. As the key engine of preindustrial transport and warfare in Eurasia, horses directly impacted the military and transport capacity of steppe societies, while long military campaigns also often required grazing areas for other livestock^63^. Together, these and other factors likely helped create an uncommonly close causal link between environmental dynamics and sociopolitical developments in the Mongolian grasslands.

The onset of humid conditions at 1200 BCE (Figs. 3, 4) coincides with drastic social changes across central Mongolia, including the first emergence of horse culture and evidence for widespread social integration across the eastern steppe. In contrast to earlier pastoralists, who were apparently constrained largely to mountain margins, DSK and other late Bronze Age herders made use of open grassland and desert regions^8,30^. At some sites, hundreds or even thousands of horse burials testify to the expanded ecological and social significance of horses^64^. The epicenter of this dramatic emergence of horse culture appears to have been central Mongolia, with large funerary and monument complexes emerging in the Khangai Mountain Range (including Zavkhan province)^65–67^. Our results suggest that the expansion of the region’s first culture, which spread as far as Trans Baikal, Tuva, Kazakhstan, Xinjiang, and China, was supported by wet conditions driven by a solar minimum.

While the NAO weakening during the grand solar minimum is associated with a general climate and environmental crisis^58^, triggering human migrations and the collapse of cultures in large parts of northern Europe^68,69^, we find the opposite causal link in Mongolia — between increased effective ecosystem moisture and positive socio-environmental impacts due to enhanced biomass production and an expansion of fertile grasslands^15,16,70^. During the grand solar minimum from 800 to 600 BCE^71^, in key social changes, and the emergence of the first integrated pastoral empires took place during a prolonged period of humid conditions, as indicated by our E_I_ and a lake water enrichment record (Δ_aq-terr_) from Lake Khar Nuur in the Mongolian Altai^30^ (Fig. 4b,e,f,h,I). Although these archives are spatially distant, they both show reduced evaporative enrichment and thus, increased effective ecosystem moisture (Fig. 4f,h)^30^. As the DSK culture waned, Mongolia witnessed an expansion of the Slab Burial culture, whose sites also yield the first direct evidence of riding tack^72^, royal equestrian burials and the earliest evidence for horsemanship appear in the archaeological record at Arzhan, in Tuva, and early mounted Scythian groups spread westward out of interior Asia^73^.

From this first expansion of horse culture, a prolonged period of humid conditions in central Mongolia supported the convergence of Mongolia’s first united pastoral polities. The Xiongnu Empire thrived particularly between 200 BCE and 100 CE (Fig. 4i)^10,74,75^, when climate conditions were also predominantly humid at Lake Telmen and Lake Khar Nuur^30^ (Fig. 4f–i). Extensive fertile grasslands favored pastoralism, while this period also saw the adoption of agriculture, the establishment of village-like settlements, increased gene flow with East and Central Asia, and extensive trade relations were established as far as the Mediterranean^6,10,12,75^. Complemented by new military and organizational techniques, climatic and environmental conditions favorable for animal pastoralism enabled the Xiongnu to form a large and powerful politically structured empire^5,8,12,32,74^.

This prolonged period of favorable climate-human interaction seems to have persisted across the early first millennium CE. This record shows that humid conditions were no guarantee of persistent political stability, as some important polities rose and fell in the Mongolian steppe. However, after the Xiongnu state failed ca. 100 CE, both the Rouran Khaganate (ca. 400 CE) and the first Turkic Khaganate (ca. 550 CE) formed during periods of favorable grassland conditions in central Mongolia^10^. This run ended with the onset of the LALIA around 600 CE (Fig. 4b–e,i). Our record shows a distinct decline in the E_I_ during this period, likely indicating cooler conditions at Lake Telmen, which is in line with previous results of Fowell et al.^24^ indicating arid conditions and the development of a cold steppe during the LALIA (Fig. 4b–e,h,i).

Just as solar minima appear to have been crucial to the first formation of pastoral empires, solar maxima may have had a disruptive effect on social integration in ancient Mongolia. Very harsh and long winters seem to have caused high livestock mortality, an increase in warfare activity, famines, and cultural re-organization during the LALIA (Fig. 4d,e,i)^5,10,27,28,33^. Based on our record (Fig. 4h), dry climate conditions prevailed during the MCA in central Mongolia, supporting previous reconstructions from central Mongolia at Lake Bayan Nuur^21^ as well as from the Mongolian Altai at Lake Khar Nuur^30^. Conditions remained unfavorable until the end of the MCA around 1300 CE. Under these conditions, failing grassland biomass may have undercut the economic and social power base of the first Turkic Khaganate, and contributed to its disintegration in ca. 603 CE^76^. During subsequent centuries, Mongolia cycled through a comparatively tumultuous period of political instability, with brief periods of steppe integration like the Second Turkic (ca. 680–740 CE) and Uyghur (ca. 750–850 CE) Khanates, interspersed with periods of domination by external powers like the Tang and Khitan states^10,36^.

Finally, our record supports previous arguments that moisture balance also played an important role in the emergence and success of the largest pastoral empire — the Great Mongol Empire of Genghis and Khubilai Khan. Our E_I_ shows a shift to humid conditions since 1100 CE and a positive effective moisture balance at the MCA-LIA transition around 1300 CE (Fig. 4h,i), which can also be seen in the Δ_aq-terr_ record from Lake Khar Nuur^30^ (Fig. 4f). This likely favored the union of nomadic tribes under Genghis Khan and the formation of the Mongol Empire, which began during the early thirteenth century and reached its greatest spatial extent during the late 13th through the mid-fourteenth century (Fig. 4i)^13,14,61^.

We conclude that solar forcing played an important role in controlling regional climate at Lake Telmen over the past 4000 years. We have shown that even small changes in temperature and precipitation have a huge impact on the effective ecosystem moisture balance and thus, biomass production and the expansion of fertile grasslands. This apparent causal link between favorable climate conditions and positive socio-environmental impacts for herding cultures in the Mongolian steppe likely had tremendous impact on the broader trajectory of human history in Eurasia, as the cyclical emergence of pastoral cultural networks and empires helped to forge some of the first pan Eurasian trade networks, spreading goods, plants, and animals, people, ideas, and even catastrophic pandemic disease^1–4^.

While these moisture fluctuations seem to have exerted an important impact on the rise and fall of Mongolian steppe cultures over the past 4000 years, in light of the paleoclimate record we expect that the near-future consequences of global warming will put the ecosystems and livelihood of the pastoral population in Central Asia at great risk. Mongolia is already experiencing a 2 °C temperature increase since 1963^77^, and will likely exceed TSI-induced temperature fluctuations in the near-future. Previous studies have shown a rapid loss of lakes^59^, melting mountain ice^78^, persistent soil moisture deficits^79,80^, and an increased frequency of droughts^79,81,82^ and heavy rainstorms^15,83,84^. Increased rainfall may not counteract the impact of rising temperatures. Instead, rainfall may exacerbate ongoing land degradation as these short-term heavy rainstorms exceed the soil’s infiltration capacity and cause surface runoff, soil erosion, and even floods^83,84^. Although, modeling results show a low probability that future drought intensities will exceed those of the last two millennia^82^, and our hydrological model suggest only a small lake level decline of 0.18 m (Supplementary Table 4) for the current temperature increase, present-day climate changes already cause enhanced socio-environmental consequences^15,81,83^, and it is uncertain whether and how modern pastoralists will to adapt to the future climate.

## Methods

### Field survey and coring

A bathymetric map of the lake basin was created with a sonar operated at a frequency of 100 kHz (Lowrance HPS 5 fish finder). The bathymetric map is based on 288,033 depth measurements and was generated in Sonar Viewer 2.1.2. Esri ArcGIS 10.5 was used for a ‘spline with barriers’ interpolation.

A 161 cm long sediment core (TL-2017/1-1) was retrieved from 22 m water depth in 2017 (48° 48′ 37.98″ N, 97° 20′ 43.9188″ E), using an UWITEC corer with hammer action (UWITEC, Mondsee, Austria).

### Sedimentological and geochemical characteristics

Detailed descriptions of determining the grain size distribution, elemental, and mineral composition are provided in the Supplementary Methods.

### Sediment core chronology

The chronology of the Lake Telmen sediment record consists of nine tie-points we identified by comparison of our evaporation index (E_I_) with the TSI record of Steinhilber et al.^44^. For each tie point, we used corresponding ages from the 22-year averaged TSI record: 1 = 1295 CE, 2 = 1141 CE, 3 = 1053 CE, 4 = 943 CE, 5 = 745 CE, 6 = 679 CE, 7 = 355 BCE, 8 = 795 BCE, 9 = 1389 BCE^44^. A Bayesian age-depth model was calculated with the package rbacon 2.4.3 in R 4.0.2^85^. All ages presented in this paper are calibrated and given as BCE (before common era) and CE (common era).

### Qualitative determination of the mineral composition

The qualitative determination of the main mineral components deduced from 13 representative samples was conducted using a Siemens D500 X-ray diffractometer (XRD) at Trier University. The quartz peak at 3.342 Å (Cu-Kα1) was accepted as internal standard for all measurements.

### Bulk isotopic composition of carbonates (δ^13^C_carb_, δ^18^O_carb_)

157 samples (ground and sieved < 40 µm) were measured with an automated carbonate-extraction device (KIELIV), coupled to a MAT253 IRMS (Thermo Fischer Scientific, Bremen, Germany) at the Helmholtz Centre Potsdam (GFZ). Up to 0.2 mg were automatically dissolved with 103% H_3_PO_4_ at 70 °C under vacuum and the isotopic composition were subsequently measured on the released and cryogenic purified CO_2_. The isotope ratios are given in delta notation against the Vienna Pee Dee Belemnite (VPDB) standard. Analytical precision was checked using replicate measurements of reference materials (NBS19, C1-internal standard), and yielded standard errors < 0.07‰ for both, δ^13^C_carb_, δ^18^O_carb_.

### *n*-alkane extraction and compound-specific δ^2^H_*n*-alkane_ measurements

Total lipids of 120 sediment samples (0.4–4.5 g) were ultrasonically extracted using a mixture of dichloromethane and methanol (9:1, v/v) as a solvent, the procedure was repeated in three cycles of 15 min each^38^. Total lipid extracts were separated by solid phase extraction using aminopropyl (Supelco; 45 µm) as stationary phase, *n*-alkanes were eluted with hexane and additionally purified over coupled silver-nitrate (AgNO_3_) coated silica gel (Supelco, 60–200 mesh) and zeolite (Geokleen Ltd.) pipette columns. Analytical measurements were performed at Friedrich Schiller University Jena. *n*-Alkane identification and quantification were performed on an Agilent 7890B gas chromatograph (Agilent, Santa Clara, California, USA) equipped with an Agilent HP5MS column (30 m × 320 µm × 0.25 µm film thickness) and a flame ionization detector (GC-FID). For identification and quantification, external *n*-alkane standards (*n*-alkane mix *n*-C_21_–*n*-C_40_, Supelco) were measured with each sequence. *n*-Alkane concentrations are given in micrograms per gram (µg g^−1^) dry weight and were calculated as the sum of *n*-C_23_ to *n*-C_35_.

δ^2^H_*n*-alkane_ analyses were performed on an isoprime visION isotope ratio mass spectrometer (Elementar, Manchester, UK) coupled via a GC5 pyrolysis–combustion interface (Elementar, Manchester, UK) to an Agilent 7890B gas chromatograph equipped with an Agilent HP5GC column (30 m × 320 µm × 0.25 µm film thickness). The GC5 operated in pyrolysis mode (ChromeHD reactor) at 1050 °C. Samples were injected in splitless mode and measured in triplicates. *n*-Alkane standards (*n*-C_27_, *n*-C_29_ and *n*-C_33_) with known isotopic composition (Schimmelmann *n*-alkane standards, Indiana, USA) were measured as duplicates after every third triplicate. The standard deviation for the triplicate measurements was < 3.6‰ for δ^2^H_*n*-C23_ and < 6.1‰ for δ^2^H_*n*-31_. However, the relatively high maximum standard deviation for δ^2^H_*n*-31_ concerns only one sample triplicate and the standard deviation was < 2.7‰ for the remaining δ^2^H_*n*-31_ triplicates. The standard deviation of standard duplicates was < 4.3‰ (n = 124). δ^2^H_*n*-alkane_ measurements were drift and amount-corrected relative to the standards in each sequence. The H3+ correction factor was checked routinely after system tuning and was stable at 4.2 ± 0.63 (n = 15). The compound-specific isotopic composition is given in delta notation versus the Vienna Standard Mean Ocean Water (VSMOW).

### The evaporation index (E_I_)

The E_I_ is based on predominantly autochthonous stable isotope values (δ^13^C_carb_, δ^18^O_carb_, and δ^2^H_*n-*C23_), which are sensitive to lake evaporation causing a distinct enrichment in ^13^C, ^18^O, and ^2^H, respectively. Since multiple isotope fractionation processes on each isotope can alter the isotopic signatures differently, all isotope values were z-transformed for standardization. The three isotopes show a similar down-core trend in terms of relative enrichment and depletion, respectively, and the E_I_ was calculated as the average of the z-standardized values.

### Water balance modeling, sensitivity analysis and model scenarios

The hydrological model J2000g adapted and extended according to the specific characteristics of closed-lake basins on the Tibetan Plateau^86^, was transferred to the Lake Telmen basin. A detailed description of the model components, model-parameter estimation and input data requirements are given in Biskop et al.^86^. As meteorological input we used climate station data (1990–2020) from Tosontsengel and lake surface water temperature from the ARC Lake data set (v3.0)^87^. To better understand the sensitivity of lake response to climate variability, we explored the effects of changes in climate input variables on several hydrological model-output components (lake evaporation, actual evapotranspiration, runoff). Lake-level changes were estimated by using the stage volume curve derived from the digital bathymetry and elevation (SRTM) elevation. Considering the paleo-lake extension of Lake Telmen, the hydrological model built for present-day conditions was run through several scenarios of precipitation and temperature changes in order to gain more quantitative knowledge about climatic conditions needed to maintain high lake-level stands during the Late Holocene. We calculated the paleo-lake extension for the + 4.8 m and + 9.4 m terrace above the present-day lake level^22^ using the water-level area curve derived from digital elevation and bathymetric data (Fig. 1).

### Applied statistics

Pearson’s correlation coefficients (r values) were calculated to identify correlations within the geochemical and stable isotope dataset. Significance of correlations were tested using a two-sided t-test (α = 0.05). For autochthonous and allochthonous endmember identification, we further calculated a principal component analysis for the elements Al, Fe, K, Mg, Na, Sr, and Ca. The applied statistic was performed with the statistical software Origin (version Pro 2019b).

## Supplementary Information

Supplementary Information.

## Data Availability

The dataset used for this study is accessible at 10.5281/zenodo.5964115.
