# Supplementary material for: Central Mongolian lake sediments reveal new insights on climate change and equestrian empires in the Eastern Steppes
Source: Sci Rep. 2022 Feb 18;12:2829. doi: 10.1038/s41598-022-06659-w (PMC8857271; doi:10.1038/s41598-022-06659-w)
Supplement: Supplementary file 1 — Supplementary Information. [file 41598_2022_6659_MOESM1_ESM.docx]

Supplementary Information for:

Central Mongolian lake sediments reveal new insights on climate change and equestrian empires in the Eastern Steppes

Julian Struck^1^*, Marcel Bliedtner^1^, Paul Strobel^1^, William Taylor^2^*, Sophie Biskop^3^, Birgit Plessen^4^, Björn Klaes^5,6^, Lucas Bittner^7,8^, Bayarsaikhan Jamsranjav^9^ Gary Salazar^10^, Sönke Szidat^10^, Alexander Brenning^3^, Enkhtuya Bazarradnaa^11^, Bruno Glaser^8^, Michael Zech^7^, Roland Zech^1^

^1^Department of Geography, Physical Geography, Friedrich Schiller University Jena, Jena, Germany

^2^University of Colorado-Boulder Museum of Natural History, Boulder, CO, USA 80309

^3^Department of Geography, Geographic Information Science, Friedrich Schiller University Jena, Jena, Germany

^4^Section Climate Dynamics and Landscape Evolution, GFZ German Research Centre for Geosciences, Potsdam, Germany

^5^Department of Geology, University of Trier, Trier, Germany

^6^Department of Soil Science, University of Trier, Trier, Germany

^7^Institute of Geography/ Physical Geography with focus on paleoenvironmental research, Technische Universität Dresden, Dresden, Germany

^8^Institute of Agricultural and Nutritional Sciences, Soil Biogeochemistry, Martin Luther University Halle-Wittenberg, Halle (Saale), Germany

^9^Max Planck Institute for the Science of Human History, Department of Archaeology, Jena, Germany

^10^Department of Chemistry, Biochemistry and Pharmaceutical Sciences and Oeschger Centre for Climate Change Research, University of Bern, Bern, Switzerland

^11^Institute of Plant and Agricultural Sciences, School of Agroecology and Business, Mongolian University of Life Sciences, Darkhan, Mongolia

* Corresponding authors: Julian Struck (julian.struck@uni-jena.de) and William Taylor (william.taylor@colorado.edu)

**S1. Chronology and age-depth modeling**

The ^14^C chronology of the sediment core TL-2017/1-1 from lake Lake Telmen is based on nine bulk TOC ^14^C ages and three compound-class (*n*-alkane) ^14^C ages, yielding a basal calibrated ^14^C age of 2300 ± 170 BCE (2,623 – 1,950 BCE (95.4%) (STab. 1). Additionally, one modern aquatic plant was dated to assess a potential ^14^C hard-water effect (Δ_HW_)^1^. This aquatic plant yielded a calibrated median age of 1828 ± 83 CE, revealing a recent Δ_HW_ of 190 ± 83 years (difference between 1828 ± 83 and 2017 CE). For age-depth modeling, Δ_HW_ was assumed to be constant over time and applied to all bulk TOC ^14^C ages. In contrast, compound-class *n*-alkane ^14^C ages were not corrected for Δ_HW_, because leaf wax homologs are dominated by long-chain *n*-C_31_-alkanes of terrestrial origin (section S3). All our ^14^C ages agree with the 95% confidence interval of this Bayesian age-depth model (ADM). The Δ_HW_-corrected bulk TOC ^14^C ages agree well with the uncorrected compound-class *n*-alkane ^14^C ages (SFig. 1). The established ^14^C chronology is stratigraphically consistent, except for three ^14^C ages. One bulk TOC ^14^C age at 21 cm is slightly too young, while another bulk TOC ^14^C age at 33 cm and one compound-class *n*-alkane ^14^C age at 42 cm appear to be too old with respect to their stratigraphic position (SFig. 1). Although the 2σ age range of these ^14^C ages overlaps with the 95% confidence interval of the Bayesian ADM, we chose to exclude them.

**
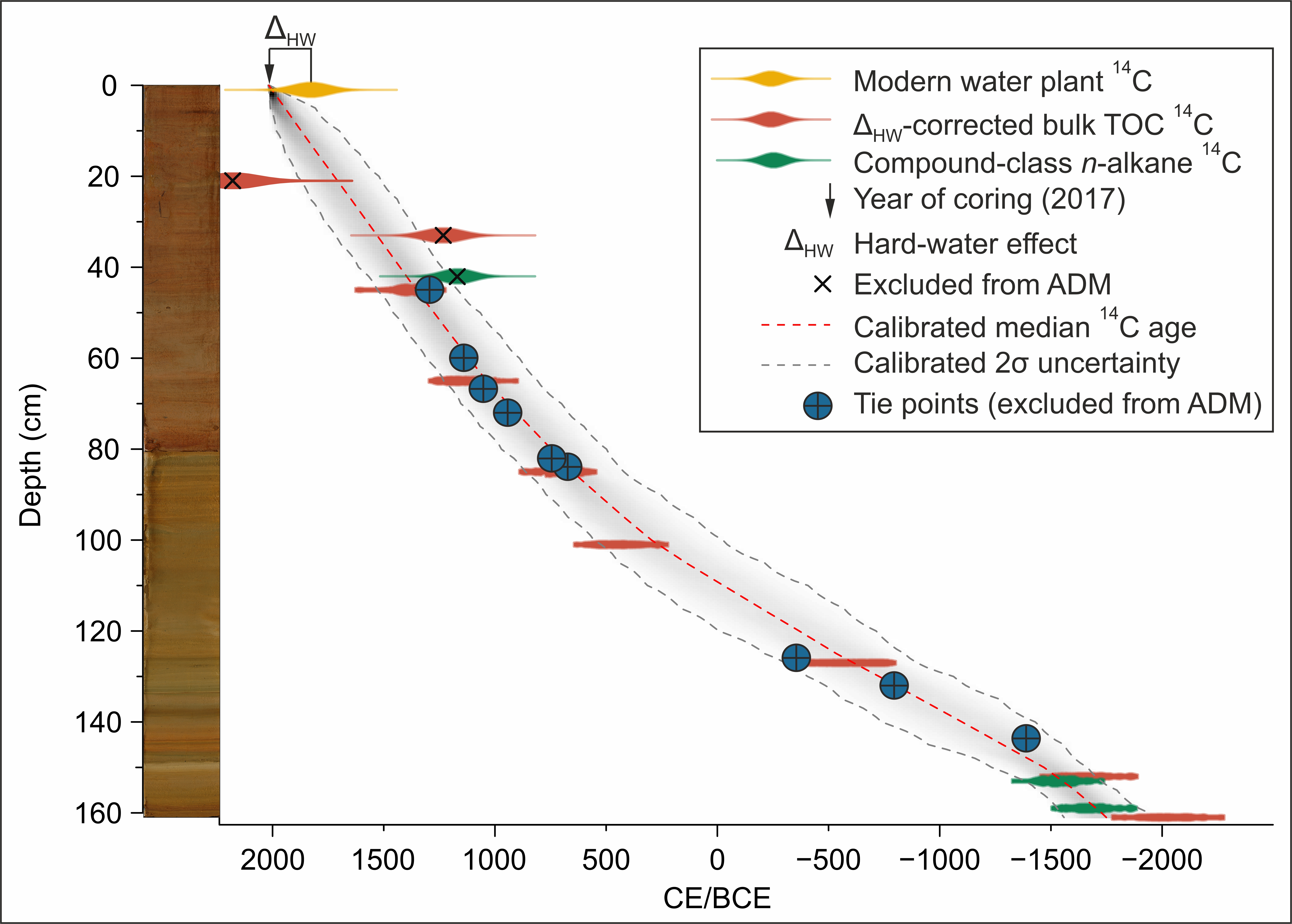
**

**Supplementary Figure 1: Photo (oxidated) of the sediment core from Lake Telmen and age-depth model (ADM).** The ADM is based on seven bulk TOC (red) and two compound-class *n*-alkane (green) ^14^C-ages. The modern hard-water effect (Δ_HW_) is the difference between the year of coring in 2017 and the ^14^C age of a modern water plant (yellow). All ages are within the 95% confidence interval of the Bayesian ADM. Tie points refer to the total solar irradiance (TSI) record published by Steinhilber et al.^2^ (SFig. 2). X = purged ^14^C ages (STab. 1; ID. 2-4).

Too young ages usually indicate mixing processes, but we can exclude these due to the stratification of the sediment core. Too old ^14^C ages could either refer to a deviating Δ_HW_ or to an input of old pre-aged carbon from the catchment^1,3,4^. However, the too old ^14^C bulk TOC age and compound-class *n*-alkane ^14^C age coincide well with each other, supporting the contribution from pre-aged carbon being more likely than Δ_HW_ variation. For the upper 50 cm of the sediment record, PC1 does not indicate an increased input of allochthonous elements from the catchment, while TOC shows increasing values (section S2). However, TOC is mainly derived from aquatic plants and algae, but a certain contribution of terrestrial pre-aged TOC is likely as indicated by the high abundance of terrestrial *n*-C_31_-alkanes within the sediment record (section S3). Thus, we suggest that pre-aged carbon from the lake’s catchment explains the slightly too old ^14^C ages in 33 and 42 cm depth, repectively^3,4^.

For further validation of the chronology, we compared the evaporation index (E_I_) with the ice core/tree ring-based 22-year average of total solar irradiance established by Steinhilber et al.^2^ (SFig. 2). Both records are in good agreement as shown by nine identified tie points. For each tie point, we used corresponding ages from the 22-year averaged total solar irradiance record (1 = 1295 CE, 2 = 1141 CE, 3 = 1053 CE, 4 = 943 CE, 5 = 745 CE, 6 = 679 CE, 7 = 355 BCE, 8 = 795 BCE, 9 = 1389 BCE)^2^. All tie points are within the 95% confidence interval of the ^14^C-based Bayesian ADM (SFig. 1). To avoid uncertainties derived from varying Δ_HW_ or contributions of pre-aged carbon, which can bias the ^14^C chronology up to several 100 years, we have established an ADM based on these nine tie points in a second iteration.

**
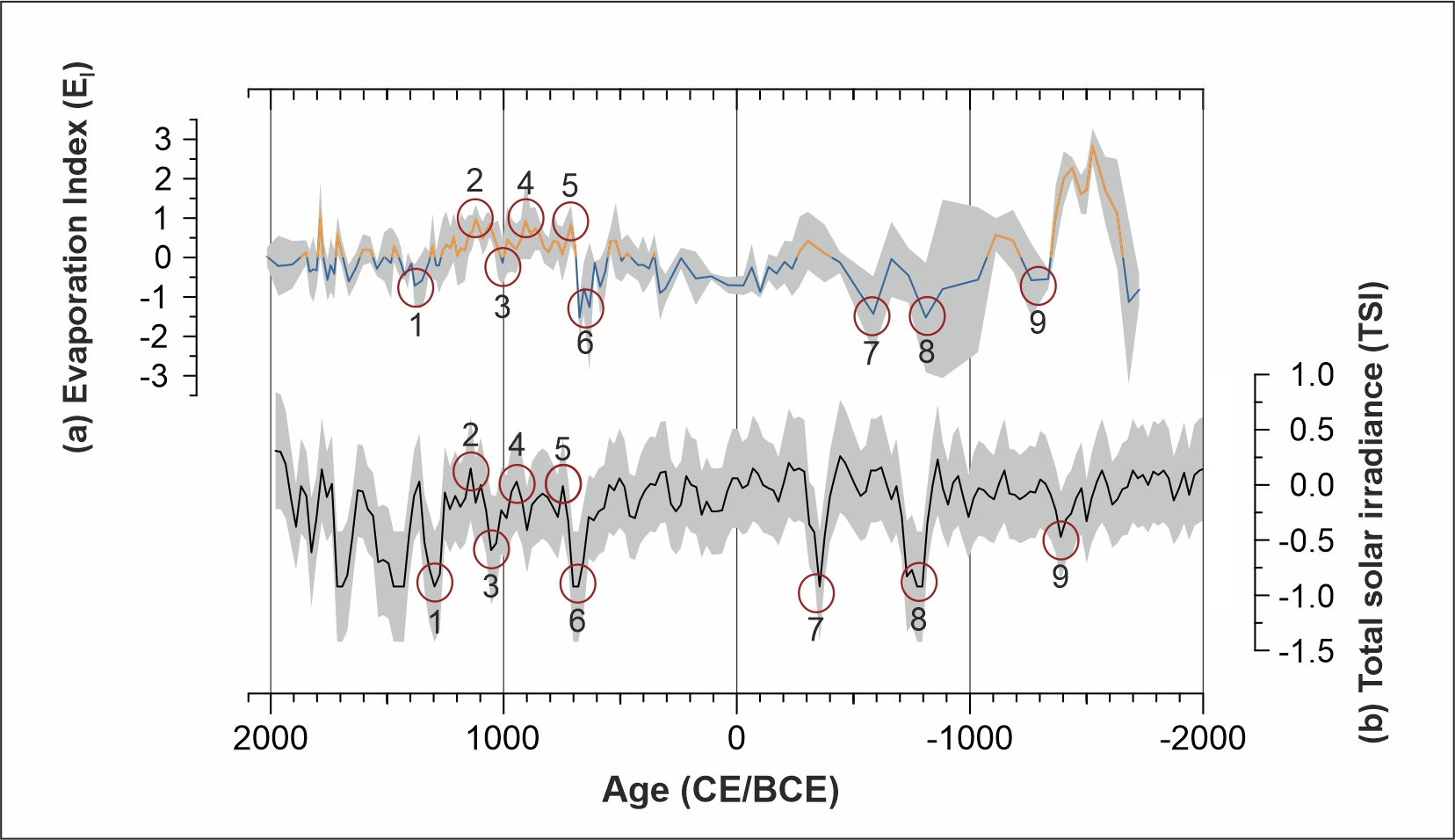
**

**Supplementary Figure 2: The evaporation index (E_I_) in comparison to the total solar irradiance record (TSI). (a)** The calculated evaporation index (E_I_) plotted against the modeled median age of the ^14^C ADM. The gray-shaded area shows the standard deviation and **(b)** total solar irradiance (TSI) given in W m^-2^ as 22-year averages. The grey-shaded area shows the 1 σ uncertainty^2^. Red circles (1-9) indicate tie-points between 2000 cal. years common era (CE) and 2000 cal. years before common era (BCE): 1 = 1295 CE, 2 = 1141 CE, 3 = 1053 CE, 4 = 943 CE, 5 = 745 CE, 6 = 679 CE, 7 = 355 BCE, 8 = 795 BCE, 9 = 1389 BCE.

**Supplementary Table 1: ^14^C calculation parameters, 2σ calibrated age ranges and calibrated median ages from Lake Telmen.** Calibrations are based on fraction modern (F^14^C) values and were performed in OxCal 4.4 using IntCal20 (ID: 1, 3-13)^5,6^ and the northern hemisphere (region 1) bomb peak (ID: 2). * marks compound-class n-alkanes ages.

| **±** | **[cal. CE/BCE]** | 83 | 93 | 90 | 73 | 103 | 132 | 82 | 149 | 143 | 150 | 110 | 119 | 171 |
| --- | --- | --- | --- | --- | --- | --- | --- | --- | --- | --- | --- | --- | --- | --- |
| **Median Age** |  | 1828 CE | 1857 CE | 1132 CE | 1173 CE | 1235 CE | 934 CE | 517 CE | 226 CE | 826 BCE | 1916 BCE | 1545 BCE | 1687 BCE | 2300 BCE |
| **Age range (2σ)** |  | 1670 - … | 1673 - … | 900 - 1286 CE | 1033 - 1276 CE | 1030 - 1405 CE | 670 - 1175 CE | 355 - 656 CE | 52 BCE - 540 CE | 1055 - 481 BCE | 2202 - 1623 BCE | 1866 -1304 BCE | 1930 -1496 BCE | 2623 - 1950 BCE |
| **±** | **[years]** | 97 | 115 | 99 | 77 | 118 | 122 | 86 | 125 | 102 | 107 | 92 | 94 | 118 |
| **age** |  | 111 | -181 | 901 | 861 | 771 | 1097 | 1539 | 1816 | 2658 | 3566 | 3264 | 3390 | 3842 |
| **δ^13^C** | **[‰]** | -68 | -26.8 | 197 | -31.8 | -25.1 | -26.3 | 185 | -24.9 | -24.1 | -28.1 | -33.4 | -35.4 | 231 |
| **u** | **[-]** | 0.014 | 0.015 | 0.011 | 0.009 | 0.013 | 0.013 | 0.009 | 0.012 | 0.009 | 0.009 | 0.008 | 0.008 | 0.009 |
| **F^14^C** |  | 1.009 | 1.023 | 0.894 | 0.898 | 0.909 | 0.872 | 0.826 | 0.798 | 0.718 | 0.642 | 0.666 | 0.656 | 0.620 |
| **Carbon mass** | **[µg]** | 128.20 | 174.40 | 196.50 | 38.00 | 157.60 | 153.50 | 184.90 | 146.70 | 125.70 | 79.20 | 61.10 | 46.90 | 230.70 |
| **Material** | **[-]** | aquatic plant | bulk TOC | bulk TOC | *n*-Alkanes* | bulk TOC | bulk TOC | bulk TOC | bulk TOC | bulk TOC | bulk TOC | *n*-Alkanes* | *n*-Alkanes* | bulk TOC |
| **Depth** | **[cm]** | [-] | 21 | 33 | 42 | 45 | 65 | 85 | 101 | 127 | 152 | 153 | 159 | 161 |
| **S. ID** | **[-]** | TL-WP | TL17-A21 | TL17-A33 | TL17-A42 | TL17-A45 | TL17-A65 | TL17-B3 | TL17-B19 | TL17-B45 | TL17-B70 | TL17-B71 | TL17-B77 | TL17-B79 |
| **B. Nr.** |  | 9760.1.1 | 9761.1.1 | 12038.1.1 | 13592.1.1 | 9762.1.1 | 9763.1.1 | 12039.1.1 | 9764.1.1 | 8912.1.1 | 8913.1.1 | 13593.1.1 | 13594.1.1 | 12040.1.1 |
| **ID** |  | **1** | **2** | **3** | **4** | **5** | **6** | **7** | **8** | **9** | **10** | **11** | **12** | **13** |

S2 Sedimentological and geochemical proxies

**Results:** The mean grain size of sediment core TL-2017/1-1 from Lake Telmen is < 32 µm and grain size distribution is clearly dominated by silt. Silt abundance ranges from 66 to 94%, while clay and sand fractions show minor contributions and range from 2 to 9% and from 0 and 29%, respectively (SFig. 3). TOC and N range from 5.4 to 12.3% and from 0.5 to 1.4%, respectively and show an increase from the bottom to the top. TIC ranges from 3.2 to 6.3% and is significantly (*α =* 0.05) anticorrelated to TOC and N (SFig. 3, STab. 2).


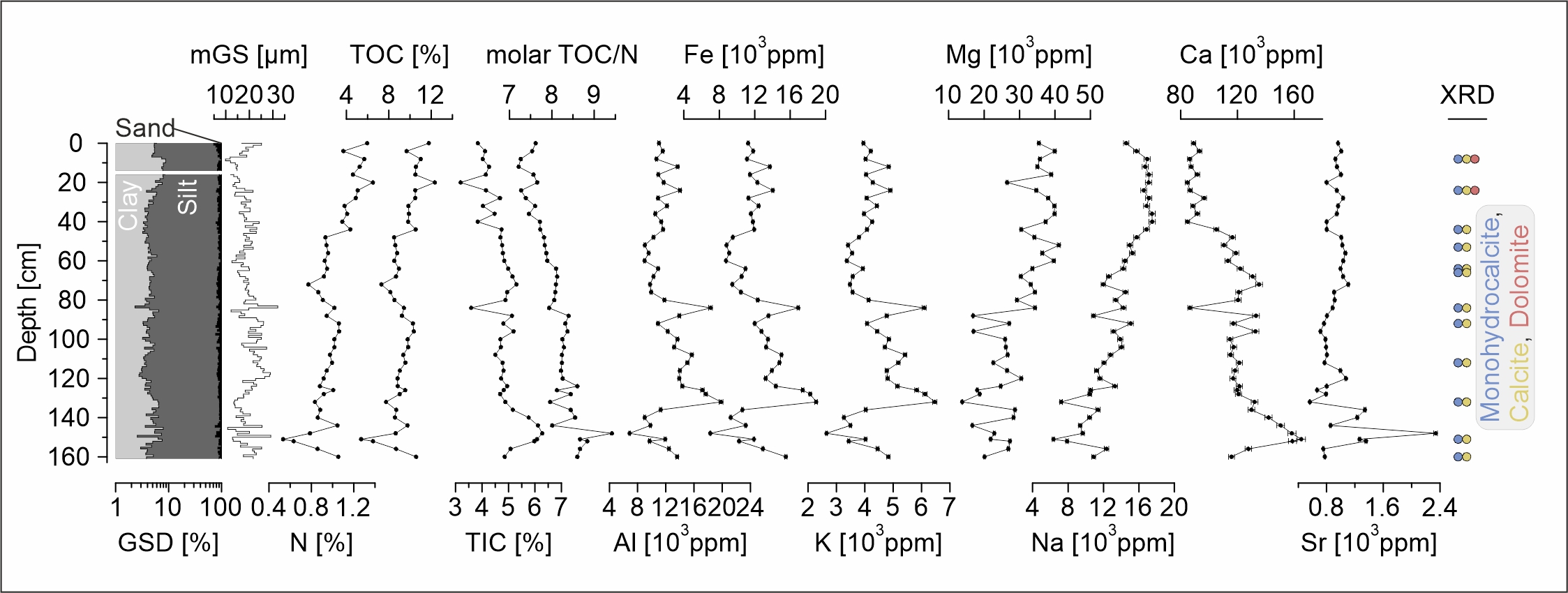
**Supplementary Figure 3: Sedimentological and geochemical results from Lake Telmen.** Depth profiles and down-core variations of grain size distribution (GSD), mean grain size (mGS), N, TOC, TIC, molar C/N, inorganic elements (Al, Fe, K, Mg, NA, Sr, Ca), and the occurring carbonate mineral forms (monohydrocalcite (blue), calcite (yellow), and dolomite (red).

The molar TOC/N ratio ranges from 7.2 to 9.4 (SFig. 3). Down-core variations of analyzed elements (Al, Fe, K, Mg, Na, Ca, Sr) enable a subdivision into three groups with a distinctly different behavior. Significant correlations (α = 0.05) were observed for i.) Al, Fe, and K, ii.) for Mg and Na, and iii.) for Ca and Sr (STab. 2). These patterns are supported by the principal component analysis (PCA) (SFig. 4). Al, Fe, and K form principal component 1 (PC1), which describes 53.3% of the total variance of the data set. PC2 describes 36.1% of the variance and includes Mg and Na (positive loadings), and also Ca and Sr (negative loadings) (SFig. 4a, b, c).

**Supplementary Table 2:** **Pearson’s r correlation matrix**. Correlation matrix of the investigated proxies Al, Fe, Ca, Mg, K, Na, Sr, TOC, TIC, N, and the molar TOC/N ratio. Bold values indicate significance (α = 0.05).

|  | Al | Fe | Ca | Mg | K | Na | Sr | TOC | | TIC | N |
| --- | --- | --- | --- | --- | --- | --- | --- | --- | --- | --- | --- |
| Fe | **1.0** |  |  |  |  |  |  |  |  | |  |
| Ca | -0.2 | -0.2 |  |  |  |  |  |  |  | |  |
| Mg | **-0.5** | **-0.5** | **-0.6** |  |  |  |  |  |  | |  |
| K | **1.0** | **1.0** | -0.2 | **-0.4** |  |  |  |  |  | |  |
| Na | -0.2 | -0.2 | **-0.9** | **0.7** | -0.1 |  |  |  |  | |  |
| Sr | **-0.6** | **-0.6** | **0.4** | 0.2 | **-0.6** | -0.2 |  |  |  | |  |
| TOC | 0.1 | 0.2 | **-0.7** | 0.2 | 0.1 | **0.7** | **-0.3** |  |  | |  |
| TIC | **-0.3** | **-0.3** | **0.9** | **-0.5** | **-0.4** | **-0.7** | **0.5** | **-0.7** |  | |  |
| N | 0.0 | 0.1 | **-0.8** | **0.3** | 0.1 | **0.8** | **-0.4** | **1.0** | **-0.8** | |  |
| molar TOC/N | 0.0 | 0.0 | **0.8** | **-0.6** | -0.1 | **-0.8** | **0.4** | **-0.5** | **0.7** | | **-0.8** |

Inferred from XRD-analyses, the mineral composition of 13 samples (SFig. 5) mainly comprises different carbonates, namely monohydrocalcite (MHC), calcite and dolomite, while we found no indication of gypsum. Dolomite only contributes to the mineral content of the two uppermost samples (SFig. 3). Salts are particularly dominated by halite. The silicate component of the samples consists of quartz, plagioclase, scarce mica (biotite, muscovite), and clays. Further differentiation of clay minerals is not possible, since XRD analyses on oriented aggregates > 2 µm have not been performed. The identified Fe-(hydr)oxides are goethite and hematite, which were summarized and represent the Fe-(hydr)oxides in Supplementary Figure 5.


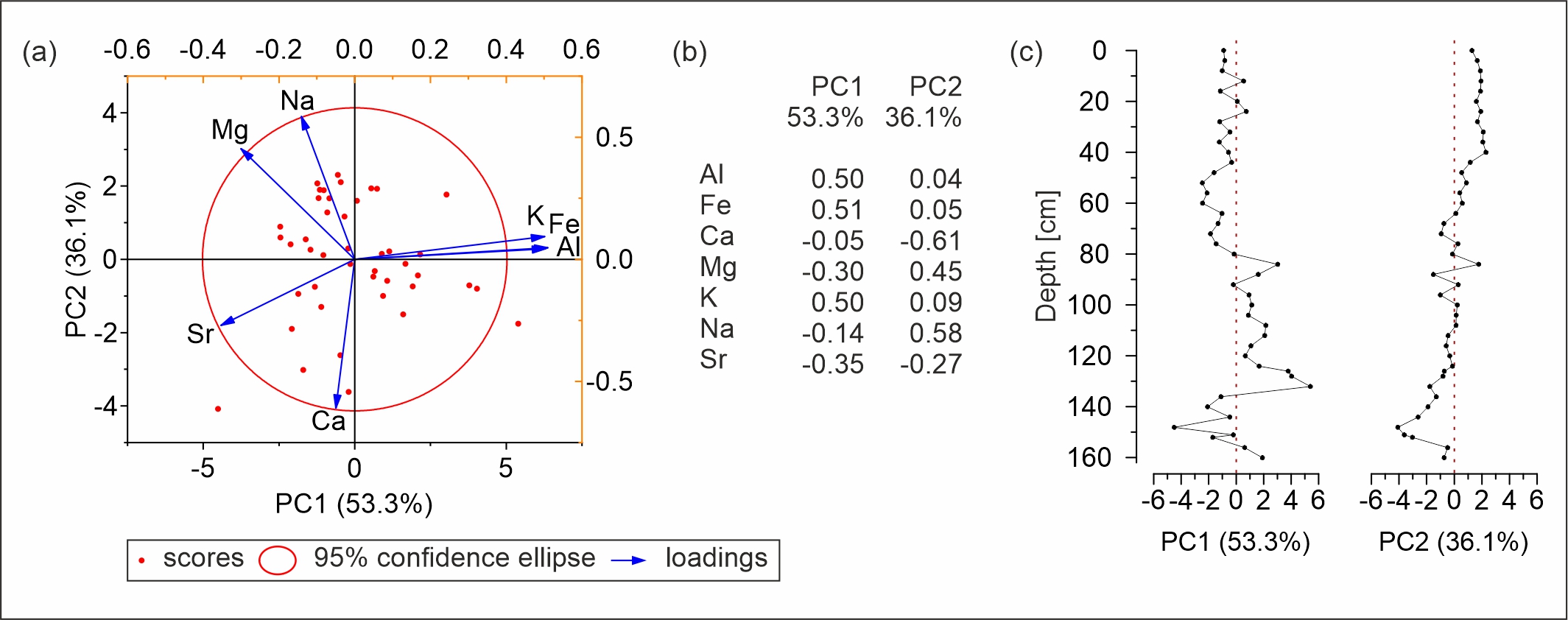


**Supplementary Figure 4: Results of the Principle Component Analysis (PCA).** PCA includes Al, Fe, Ca, Mg, K, Na, and Sr as variables. **(a)** Biplot of PC1 and PC2, **(b)** loading values for each variable of PC1 and PC2 **(c)** depth profiles, and down core score variations of PC1 and PC2.

**
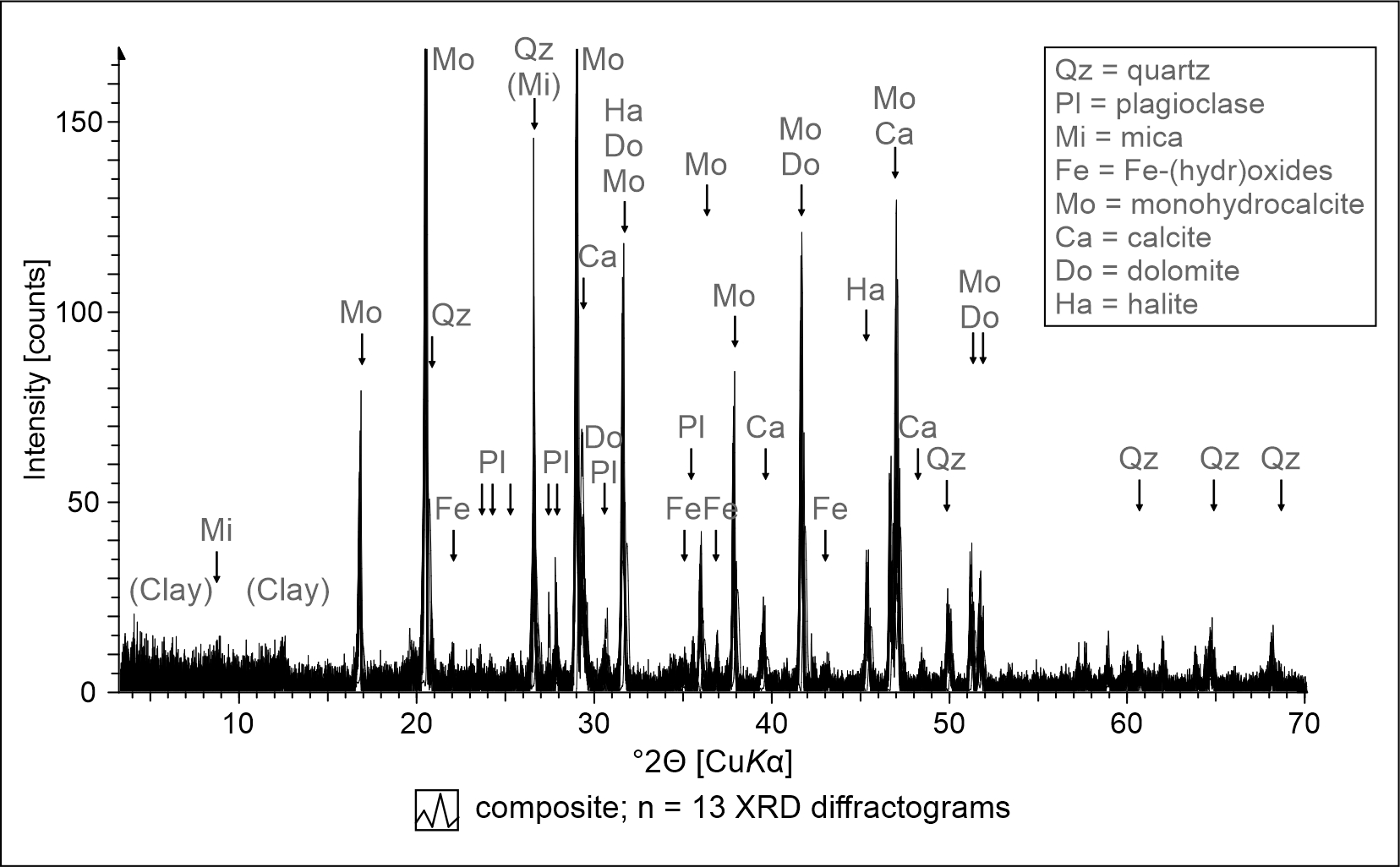
**

**Supplementary Figure 5:** **Results of the XRD analyses performed on 13 representative samples.** The individual diffractograms were compiled and are presented as composite.

**Interpretation:** The PCA allows differentiation of provenance and deposition in terms of dominant transport mechanisms. PC1 is loaded by Al, Fe, and K and indicates allochthonous sediment contributions from the catchment (SFig. 4a, b, c). This is supported by XRD-analyses, documenting Fe, Al, and K-bearing mineral phases, such as silicates, Fe-(hydr)oxides, and clays (SFig. 5). We attribute a certain amount of silt-sized quartz to a wind-borne deposition, whereas Fe-(hydroxides) and other silicate components are mostly inputs due to chemical weathering and physical erosion processes in the catchment^7–10^. Autochthonous production/precipitation is represented by PC2. Positive scores of PC2 include Mg and Na, pointing to higher in-situ salt precipitation^11^. However, a certain contribution of Mg and Na of allochthonous-derived plagioclase and mica cannot be excluded and might contribute to the Mg and Na concentrations in the sediments of Telmen Nuur. By contrast, negative scores of PC2 include Ca and Sr – associated with pronounced carbonate precipitation (SFig. 4a, b, c). Significant correlations between TIC, Ca, and Sr (STab. 2) indicate a Sr-fixation by calcifying organisms^12^. The variability of occurring carbonates indicates MHC, calcite, and dolomite (SFigs. 3, 5). Calcite and dolomite content may also reside from an allochthonous source, whereas MHC clearly indicates an autochthonous endmember in lake sediments, and its precipitation is either chemical and/or biogenic^13–15^. In a previous study, Peck et al.^16^ described the formation and rapid accumulation of well-formed Ca-aggregates and calcite crystals during the productive period in summer, primarily reflecting a biogenic origin. These Carbonate deposits alternate with amorphous TOC accumulations, which settled from the water column during the non-productive period (from fall to spring)^16^. In Lake Telmen, TOC is predominantly derived from aquatic plants and algae as indicated by relatively low molar TOC/N ratios^17–19^ (SFig. 4).

S3. *n*-Alkanes and compound-specific δ^2^H*_n_*_-alkane_ and δ^13^C*_n_*_-alkane_

**Results and interpretation:** In the sediment record from Lake Telmen, total *n*-alkane concentration (*n*-C_23_ - *n*-C_35_) ranges from 4 to 87 µg g^-1^ sediment dry weight. The *n*-alkane distribution is dominated by *n*-C_31_-alkanes (20 - 33%), the contribution of *n*-C_23_-alkanes is distinctly lower and contributes only to 5 to 16% of the total *n*-alkane concentration (SFig. 6). *n*-C_31_-Alkanes are mainly synthesized by terrestrial plants and originate from the lake’s catchment^20,21^, while *n*-C_23_-alkanes are synthesized in higher amounts by submerged and floating aquatic plants^21–23^. A terrestrial contribution to the *n*-C_23_-alkane pool cannot entirely be excluded, however, previous investigations have shown that *n*-C_23_-alkane does not represent the dominant homologs in the plants and respective topsoils from the lake’s catchment^21^. They contribute less than 5% of the total *n*-alkane concentrations from the prevailing plants, including *Poaceae, Cyperaceae,* *Artemisia* spp., and *Caragana* spp.^20^. Therefore, we interpret *n*-C_31_ originating from the terrestrial system and *n*-C_23_ being predominantly synthesized in this lacustrine environment. These two endmembers (allochthonous vs. autochthonous) are well reflected in the compound-specific isotopic signatures of Lake Telmen (SFig. 6), indicating different δ^2^H*_n_*_-alkane_ ranges and trend behaviors for the *n*-C_23_ and *n*-C_31_-alkanes.

The δ^2^H*_n_*_-alkane_ signatures of terrestrial *n*-C_31_-alkanes (δ^2^H*_n_*_-C31_) record mainly the isotopic signature of precipitation (δ^2^H_p_), whereas δ^2^H*_n_*_-alkane_ signatures of aquatic *n*-C_23_-alkanes (δ^2^H*_n_*_-C23_) mainly mirror the isotopic signature of lake water (δ^2^H_LW_)^23–26^. However, numerous fractionation processes (e.g., biosynthetic fractionation) can alter the δ^2^H*_n_*_-alkane_ signatures, and evapotranspiration and lake water evaporation can cause a distinct ^2^H enrichment^25,27,28^. Apparent fractionation (ε*_n_*_-alkane/p_), enclosing the difference between δ^2^H_p_ and δ^2^H*_n_*_-alkane_, integrates over all possible fractionation processes and evapotranspirative effects and results in ^2^H depleted δ^2^H*_n_*_-alkane_ values relative to δ^2^H_p_^25,27^. Struck et al.^29^ concluded that apparent fractionation for *n*-C_31_-alkanes remains relatively constant along a Mongolian topsoil transect (-146 ± 14‰) and reflects δ^2^H_p_ with a root-mean-square error of 12.6‰. A distinct anticorrelation between the δ^2^H*_n_*_-C31_ values and δ^18^O values from Dongge Cave suggests an underlying influence of atmospheric circulation (section S7)^30^. By contrast, the *n*-C_23_-alkatone is consistent with δ^2^H_LW_ and is therefore very sensitive for lake water evaporative ^2^H enrichment^23,24,28^.

**
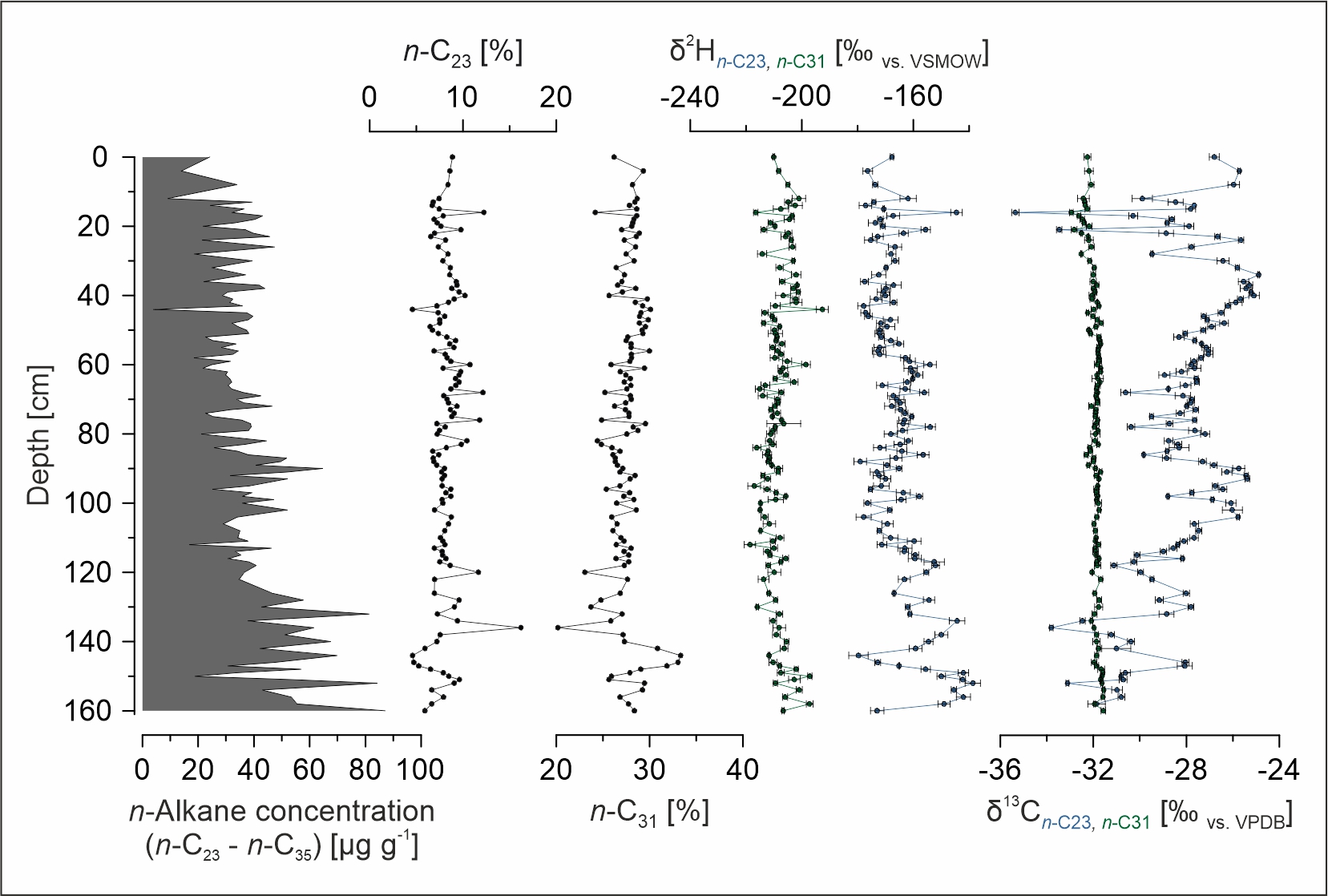
**

**Supplementary Figure 6: *n*-Alkane results and compound-specific isotopes from Lake Telmen.** Depth profiles and down core variations of the total *n*-alkane concentration (*n*-C_23_ – *n*-C_35_), percentage of *n*-C_23_ and *n*-C_31_-alkanes, δ^2^H*_n_*_-C23_ (blue graph), and δ^2^H*_n_*_-C31_ (green graph).

Apart from δ^2^H*_n_*_-alkane_, we additionally analyzed the compound-specific δ^13^C*_n_*_-alkane_ signatures which range from -32.9 ± 0.1 to -31.6 ± 0.1‰ for δ^13^C*_n_*_-C31_ and from -35.4 ± 0.2 to -24.9 ± 0.0‰ for δ^13^C*_n_*_-C23_, respectively (SFig. 6). δ^13^C*_n_*_-C31_ values are clearly related to C_3_ plants^31^, which is consistent with δ^13^C*_n_*_-alkane_ signatures from the lake’s catchment^20^. Thus, δ^13^C*_n_*_-C31_ values predominantly indicate the photorespiration of C_3_ plants, which is mainly affected by the process of water use efficiency (WUE), causing a ^13^C enrichment under distinctly arid conditions^18,20,31^. However, δ^13^C*_n_*_-C31_ values are nearly constant at ~32‰ and down core variations of 1.4‰ are quite small and should not be overinterpreted.

Since *n*-C_23_-alkanes are predominantly of aquatic origin, δ^13^C*_n_*_-C23_ isotopes primarily reflect aquatic bio productivity within the lake’s system and the assimilation of different carbon sources during the synthesis of *n*-C_23_-alkanes (dissolved CO_2_ vs. dissolved bicarbonate (HCO_3_^-^)). Relatively ^13^C-enriched *n*-C_23_-alkanes compared to the constant δ^13^C*_n_*_-C31_ signatures indicate enhanced assimilation of dissolved HCO_3_^-^ (δ^13^C = 1‰) and a limitation of dissolved CO_2_ (δ^13^C = -7‰), which cause a distinct ^13^C enrichment in the *n*-alkanes^17,19,32^. Possible control factors influencing these processes are an increase in aquatic bio productivity (macrophytes, algae, etc.) due to higher temperatures, an increase in nutrient supply^21,33^ and/or a prolonged ice cover at the site that inhibits atmospheric CO_2_ exchange^21,34^.

S4. Hemicellulose-derived sugar fucose and compound-specific δ^18^O_Fucose_

The hemicellulose-derived sugar fucose was present in all analyzed samples in sufficient amounts for δ^18^O_Fucose_ measurements. Fucose is predominantly synthesized by aquatic plants and therefore δ^18^O_Fucose_ values reflect variations in the isotopic signature and evaporative ^18^O enrichment of lake water^35^. Compound-specific δ^18^O_Fucose_ values range from 31.8 ± 0.2 to 38.5 ± 1.4‰. Although δ^18^O_Fucose_ shows a similar down-core trend in comparison to the analyzed δ^13^C_carb_, δ^18^O_carb_, and δ^2^H*_n_*_-C23_ values (SFig. 7), we have excluded δ^18^O_Fucose_ from the E_I_ calculations due to much lower sample density.

S5. Evaporation index (E_I_)

δ^13^C_carb_, δ^18^O_carb_, and δ^2^H*_n_*_-C23_ values reveal similar down-core trends and a distinct isotope enrichment during phases of higher autochthonous production, associated with warm and dry conditions and enhanced lake water evaporation (SFig. 7). While δ^18^O_carb_ and δ^2^H*_n_*_-C23_ values directly refer to the isotopic signature and evaporative ^18^O and ^2^H enrichment of lake water, δ^13^C_carb_ values refer to bio productivity, decomposition of organic matter, and atmospheric CO_2_ exchange^24–26,28,34,36–40^. The highly significant covariation between autochthonous δ^13^C_carb_ and δ^18^O_carb_ (r = 0.61, *p* = 1.06e^-17^), reflect equilibrium conditions of dissolved and atmospheric CO_2_, which is highest between 130 and 161 cm core depth (r = 0.86, p = 4.64e^-7^)^36,38,39^. Therefore, both isotopes of autochthonous carbonates indicate a sensitive response to lake evaporation. Enhanced evaporation increases the CO_2_ partial pressure and favors the exchange of ^12^CO_2_, causing a significant enrichment^34,36,38,39^. An additional modification of δ^13^C_carb_ through the assimilation of ^13^C-enriched bicarbonate seems to be possible.

Although similar down core trends and covariation of δ^18^O_carb_ and δ^2^H*_n_*_-C23_ are significant, the r-value is quite low (r = 0.19, p = 0.04). Probable reasons for these deviations are, among others:

1. **Contributions of allochthonous material:** For δ^13^C_carb_ and δ^18^O_carb_ a certain bias from allochthonous carbonates cannot be excluded completely, particularly in the uppermost part of the sediment core where dolomite has been detected (SFig. 2)^7,9^.
2. **Different mineral phases of carbonates:** MHC and calcite were identified as the most abundant carbonate phases in Lake Telmen sediments (SFigs 3; 5). Li et al.^13^ reported more enriched δ^13^C_carb_ and δ^18^O_carb_ values for bulk carbonate samples consisting of MHC and calcite, than for samples consisting of calcite only. Dolomite is generally more enriched in ^18^O which leads to ~3‰ more positive δ^18^O_carb_ values compared to calcite^40^.
3. **Variations in biosynthetic fractionation:** δ^18^O_carb_ might be affected by different, unknown (biosynthetic) fractionation factors, probably related to the occurrence of multiple ostracod species in Lake Telmen^41^. The δ^2^H*_n_*_-C23_ values are also affected by biosynthetic fractionation processes, and values of -160‰ and -105‰ were suggested previously for aquatic *n*-alkanes^42–46^.
4. **Salinity-dependent fractionation processes:** Since Lake Telmen is a hyposaline oligotrophic lake, an influence on isotope values modified by variations of lake water salinity is expected. For δ^2^H*_n_*_-C23_, previous studies propose a ^2^H depletion with increasing salinity (2.6‰ per salinity unit (1g l^-1^))^47^. However, investigations on regional diatoms revealed only minor salinity fluctuations for the past ~4000 years, ranging between 2 and 4 g l^-1 48^. Therefore, we suggest that the effect of lake water enrichment is stronger than a possible ^2^H depletion caused by salinity changes.


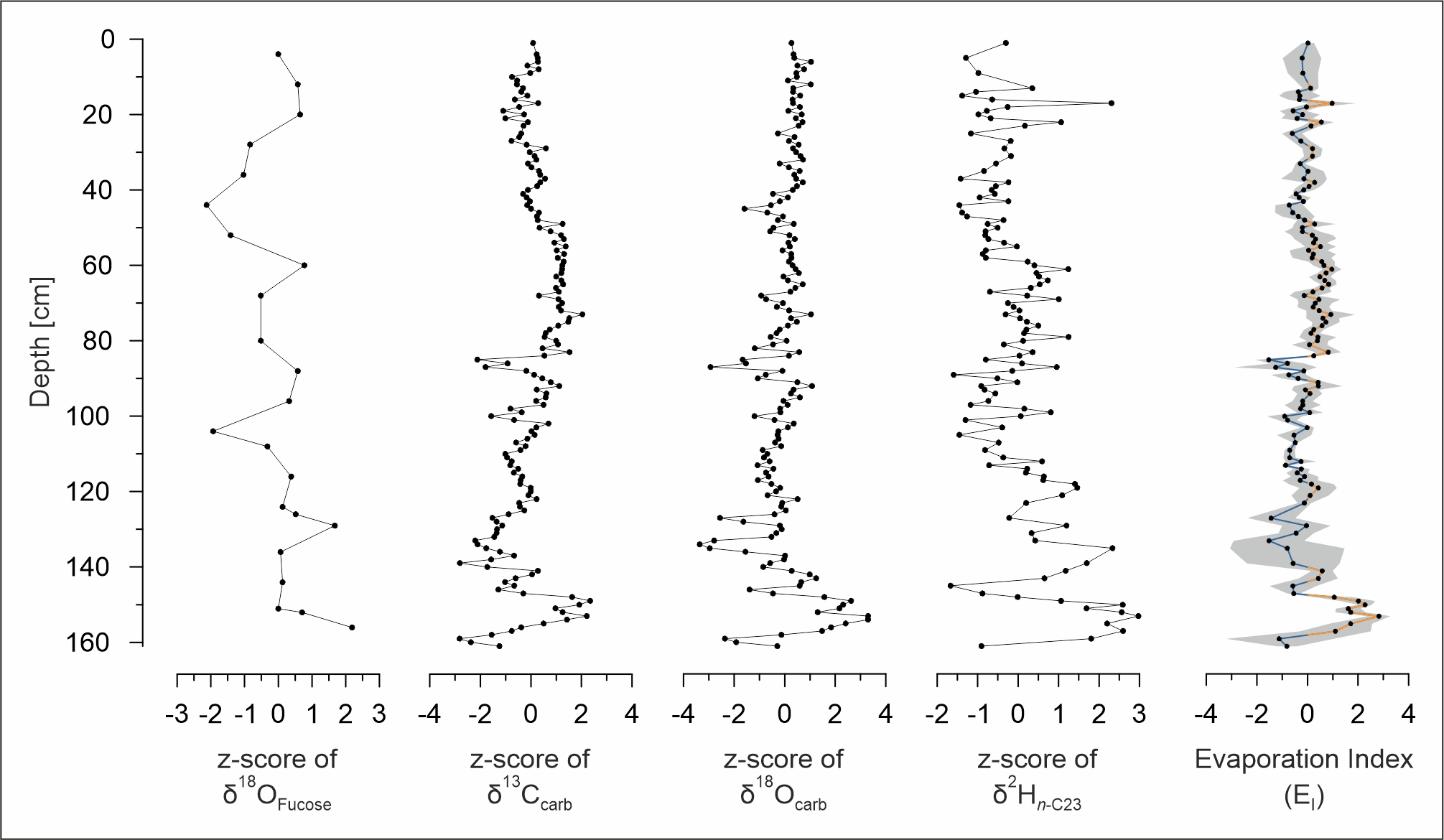


**Supplementary Figure 7: Z-score normalized isotope data and the calculated evaporation index (E_I_) from Lake Telmen.** Depth profiles and down-core variations of normalized (z-scored) isotope values (δ^18^O_Fucose_, δ^13^C_carb_, δ^18^O_carb_, and δ^2^H_n-C23_) and the E_I_ (gray-shaded area = standard deviation). Positive E_I_ values (yellow) refer to dry conditions, negative values (blue) refer to humid conditions.

Despite existing limitations and uncertainties, the four isotopes clearly show similar down core trends and a sensitive response to lake water evaporation. Therefore, we have normalized the isotope data by z-transformation and proposed an averaged evaporation index based on δ^13^C_carb_, δ^18^O_carb_, and δ^2^H*_n_*_-C23_ for further paleoclimatic interpretation (SFig. 7).

S6. Present-day water balance model and water-balance sensitivity

The model run for present-day conditions (1990 – 2020) captures mean annual estimates of single water balance components (STab. 3) reasonably well with respect to findings of Minderlein et al.^49^ and Nandintsetseg et al.^50^, investigating evapotranspiration and water balance dynamics in Mongolia.

**Supplementary Table 3: Present-day long-term average water-balance components and water budget for the Lake Telmen basin.**

| Long-term average water balance components (mm yr^-1^) | | | | | | |
| --- | --- | --- | --- | --- | --- | --- |
| P_lake_ | E_lake_ | | P_land_ | AET_land_ | | R_land_ |
| 235 | 612 | | 258 | 236 | | 22 |
| Long-term average water budget (km³ yr^-1^) | | | | | | |
| Water loss (Net E_lake_ = P_lake_ - E_lake_) | | Water gain (R_land_ = P_land_ - AET_land_) | | | Net water-budget (∆V_lake_ = Net E_lake_ + R_land_) | |
| -0.075 | | 0.075 | | | -1.0e^-4^ | |

P_lake_ = on-lake precipitation, E_lake_ = lake evaporation, P_land_ = precipitation over land, AET_land_ = actual evapotranspiration, R_land_ = runoff from land surface, Net E_lake_ = net evaporation, ∆V_lake_ = lake-volume change

Various sensitivity model runs reveal high sensitivity of several water-balance components to climate variations (STab. 4). A 5-10% increase (decrease) in precipitation leads to a modeled increase (decrease) in mean annual lake level by 0.11-0.20 m (0.09-0.14 m) compared to present-day conditions. This is primarily caused by an increase (decrease) in runoff of ~25-50% (~25-30%). Higher and lower precipitation amounts result in increasing or decreasing AET (~2.5-5 vs. ~3-7%), which may reduce the impact of changing precipitation on lake-level variations. Thus, variations of air temperature of 1-2°C cause a lake evaporation change of ±4.5-9%, an AET increase (decrease) of ~2-3% (~2.5-6%), and a runoff increase (decrease) of ~25-65% (~20-35%), leading to a lower (higher) lake-level of ~0.1-0.2 m (~0.1-0.3 m) relative to present-day conditions. Increasing (decreasing) lake surface temperature (1-2°C) results in a lake evaporation increase (decrease) of ~6% (~8-9%) (STab. 4).

**Supplementary Table 4: Absolute (percentage) changes in water-balance components and lake-level changes by increasing/decreasing input variables.**

|  |  | E_lake_ [mm] / [%] | AET_land_ [mm] / [%] | R_land_ [mm] / [%] | Lake level [m] |
| --- | --- | --- | --- | --- | --- |
| Precipitation | +5% | - | +6/+2.5 | +6/+27 | +0.11 |
|  | +10% | - | +12/+5.1 | +11/+50 | +0.20 |
|  | -5% | - | -7/-3.0 | -5/-23 | -0.09 |
|  | -10% | - | -16/-6.9 | -7/-32 | -0.14 |
| Air temperature | +1°C | +28/+4.6 | +5/+2.1 | -5/-23 | -0.11 |
|  | +2°C | +56/+9.2 | +8/+3.4 | -8/-36 | -0.18 |
|  | -1°C | -28/-4.6 | -6/-2.5 | +6/+27 | +0.13 |
|  | -2°C | -56/-9.2 | -14/-5.9 | +14/+64 | +0.28 |
| Lake surface temperature | +1°C | +36/+5.9 | - | - | -0.03 |
|  | +2°C | +39/+6.4 | - | - | -0.04 |
|  | -1°C | -48/-7.8 | - | - | +0.05 |
|  | -2°C | -57/-9.3 | - | - | +0.05 |

E_lake_ = lake evaporation, AET_land_ = actual evapotranspiration, R_land_ = runoff from land surface

S7. The influence of atmospheric circulation systems on regional climate?

In addition to the regional paleohydrological changes revealed by the E_I_ (Fig. 2b), the δ^2^H*_n_*_-C31_ values refer to the isotopic signature of precipitation in Mongolia^29^ but so far, underlying mechanisms remain uncertain for this region. Based on a modern δ^2^H*_n_*_-C31_ calibration^29^, an amount or temperature effect can be excluded in Mongolia. Therefore, a distinct anti-phasing between δ^2^H*_n_*_-C31_ and Dongge Cave δ^18^O (SFig. 8a, c) suggests an underlying influence of atmospheric circulation systems regarding different precipitation sources (Westerlies vs. EASM). More positive δ^2^H*_n_*_-C31_ values at Lake Telmen and more negative δ^18^O values at Dongge Cave^30^ are prominent during the drier early Late Holocene (SFig. 8a, c). The latter is interpreted to document a strong EASM^30^, which is in agreement with more humid conditions, forest-steppe development, and dune stabilization in the Mu Us Desert in Inner Mongolia (China), during the early Late Holocene^51^. The strong EASM, however, did not reach Lake Telmen during the early Late Holocene. Instead, the subsidence zone North of the monsoon region seems to have intensified and/or shifted northward^51^, causing enhanced aridity and evaporative enrichment at Lake Telmen. A stronger influence of the EASM on central Mongolia was recently suggested by Dugerdil et al.^52^. However, their brGDGT and pollen-based results suggest humid conditions during the early Late Holocene, contradicting the results from Lake Telmen and the hypothesis of a distinctly northward-shifted subsidence zone.


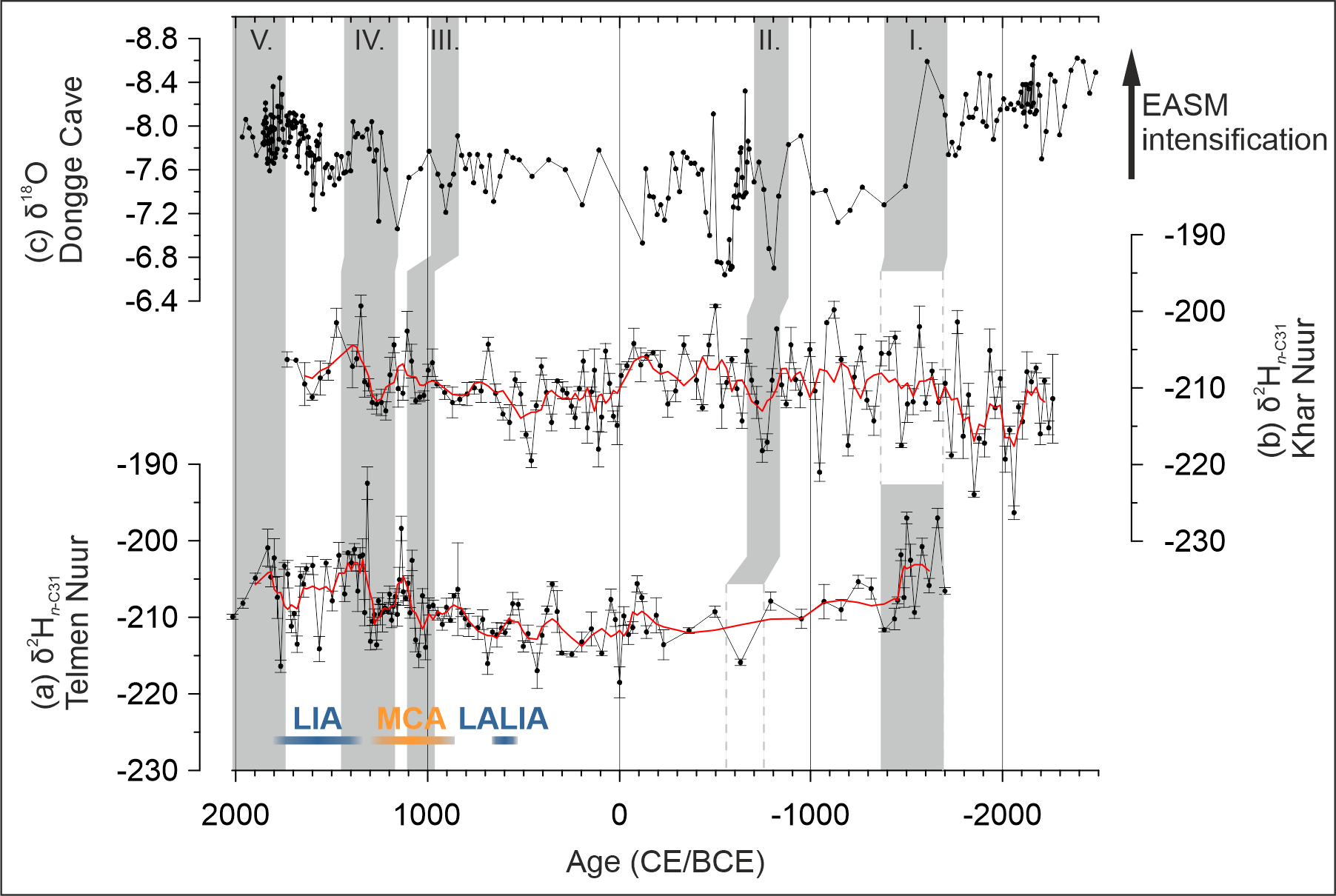


**Supplementary Figure 8: Comparison of δ^2^H_n-C31_ records from Mongolia. (a)** δ^2^H_n-C31_ values from Lake Telmen. Red line = moving average over 5 positions. **(b)** δ^2^H_n-C31_ values from Lake Khar Nuur^53^. Red line = moving average over 5 positions. **(c)** δ^18^O values from Dongge Cave^30^**.** All isotope values (a, b, c) are given in permille [‰] vs. Vienna Standard Mean Ocean Water (VSMOW). Gray bars (I. – V.) show the anti-phasing between Mongolian δ^2^H_n-C31_ records and the Dongge Cave δ^18^O record. Climate anomalies: Late Antique Little Ice Age (LALIA), Medieval Climate Anomaly (MCA), and Little Ice Age (LIA) are shown after Büntgen et al.^54^.

The δ^2^H*_n_*_-C31_ trend from Lake Telmen is slightly inverse compared to the δ^2^H*_n_*_-C31_ record from Lake Khar Nuur^53^ until 250 BC (SFig. 8a, b). This might indicate a turning point in the respective forcing system because thereafter, both records show the same δ^2^H*_n_*_-C31_ trends during the course of the Common Era (i.e., the past 2000 years). This turning point is identical to the “climate system dynamic” reconstruction of Dugerdil et al.^52^, indicating a shift from an EASM-dominated system to a Westerly-dominated system in central Mongolia and the Khangai region at 2250 cal. ka BP (250 BCE), while the Mongolian Altai was entirely controlled by the Westerlies during the course of the Late Holocene^52^. At Khar Nuur, δ^2^H*_n_*_-C31_ values are not strikingly more positive during the early Late Holocene, and thus, the record reveals no anti-phasing with the Dongge Cave δ^18^O record^30,53^ (SFig. 8b, c.). However, during the grand solar minimum around 800 BC^2^, the intensity of the EASM decreases, which coincides with negative δ^2^H*_n_*_-C31_ values in the Khar Nuur record^53^ (SFig. 8b). Due to the lower sampling density, this trend depression cannot clearly be detected in the Lake Telmen record. Previous investigations suggest an increased influence of the mid-latitude Westerlies around 1000 BCE^55,56^, which is associated with enhanced sediment relocation and geomorphological activity in central Mongolia, indicating more humid conditions^57–59^. Ongoing from ∼250 BCE, the records from Lake Telmen and Lake Khar Nuur reveal similar trends showing more negative δ^2^H*_n_*_-C31_ values during a period of reduced EASM intensity^30^ (SFig. 8). Especially between the MCA and the LIA, both records show a decrease towards more negative δ^2^H*_n_*_-C31_ values, which coincides with an abrupt EASM weakening observed by Lan et al.^60^. Moreover, Lan et al.^60^ have shown a northward shift of the EASM during the MCA, which likely affected the climate in central Mongolia due to an intensified and northward-shifted subsidence zone. Drier conditions during this period coincide with severe droughts in central Mongolia previously reported by Pederson et al.^61^ and distinct vegetation changes in the Mongolian Altai^62^. By contrast, the climate during the LIA is generally associated with more humid conditions and again, intensified Westerlies^56,61,62^. Our δ^2^H*_n_*_-C31_ record from Lake Telmen indicates a certain influence of atmospheric circulation and thus, might allow to disentangle the influences of the EASM and the Westerlies, and thus, an isotopic identification of the precipitation source. However, this approach needs further comparison to direct precipitation proxies and validation on archives spanning the entire Holocene, where especially EASM fluctuations were more prominent.

S8. Supplementary Methods

**^14^C measurements, chronology, and age-depth modeling:**

We have analyzed nine bulk TOC and three compound-class *n*-alkane ^14^C samples. In addition, a modern water plant was dated to evaluate a potential hard-water effect. The ^14^C analyzes were carried out on a MIni CArbon DAting System (MICADAS) AMS coupled online to an Elementar Analyzer (Elementar, Manchester, UK) at the LARA AMS Laboratory of the University of Bern, Switzerland^63^. For bulk TOC ^14^C measurements, freeze-dried (-50 °C; >72h) and ground sediments were treated with 1M hydrochloric acid (HCl, 10%) at 60 °C for 12 hours to remove carbonates, rinsed to pH neutrality and packed into tin boats (6 * 6 * 12). For compound-class dating, purified non-heated *n*-alkane fractions (*n*-C_23_ - *n*-C_35_) were transferred with dichloromethane (HPLC Grade) into tin capsules (3.5 * 5.5 * 0.1). Results are reported as fraction modern (F^14^C), which is the activity ratio of a sample related to the modern reference material Oxalic Acid II after subtracting the background signal (STab. 1). F^14^C results from the LARA AMS were corrected for cross (carry-over effects) and constant contamination (carbon mass and F^14^C of the tin boats/caps) according to the contamination drift model of Salazar et al.^64^. For constant contamination, ten tin boats and ten capsules were joined and measured which yielded 2.16 and 0.38 µg C for a single boat/cap with F^14^C values of 0.521 and 0.557.

^14^C ages were calibrated using the IntCal20 calibration curve^5,6^. The ^14^C chronology of the Lake Telmen sediment record consists of seven hard-water-corrected bulk ^14^C TOC ages and two compound-class *n*-alkane ^14^C ages. The Hard-water effect was evaluated by the ^14^C results of the modern water plant, which yielded an uncalibrated age of 111 ± 97 years. A Bayesian age-depth model was calculated with the package rbacon 2.4.3 in R 4.0.2^65^. All ages presented in this paper are calibrated and given as BCE (before common era) and CE (common era).

**Grain size distribution:** The grain size distribution was measured with a Laser Diffraction Particle Size Analyzer (LS 13320, Beckman Coulter, California, USA) in 1 cm resolution at the Friedrich Schiller University Jena. Samples were sieved < 2mm and treated with hydrogen peroxide (H_2_O_2_; 15%, 30%) and HCl (10%), to remove organic matter and carbonates, respectively. Before each measurement, samples were shaken for two hours. Measurements were performed with the Aqueous Liquid Module in several cycles (60 sec.). The ‘Fraunhofer’ optical model was used for computing the grain size distribution. Granulometric parameters were calculated from the first reproducibility signal with Gradistat 4.2 software.

**Elemental composition:** The elemental composition (Al, Fe, Ca, Mg, K, Na, Sr) of 43 samples were measured with an ICP-OES (725-ES, Varian, California, USA) at the Friedrich Schiller University Jena. Before the measurement, samples were freeze-dried (-50 °C; >72h), ground, and sieved to <40 µm and ~0.2 g were dissolved in a microwave-assisted modified aqua regia digestion (2 ml HCl (32%), 4 ml HNO_3_ (65%)). Relative errors were determined based on triplicate measurements (relative error (n = 2): Al ≤ 1.42%, Fe ≤ 0.82%, Ca ≤ 1.78%, Mg ≤ 1.11%, K ≤ 1.17%, Na ≤ 2.09%, Sr ≤ 1.11%) and analytical errors were determined based on reference material LGC6 187 (relative error (n = 9): Al ≤ 1.76%, Fe ≤ 0.45%, Ca ≤ 0.86%, Mg ≤ 0.68%, K ≤ 2.01%, Na ≤ 13.52%, Sr ≤ 0.69%).

**Carbon and Nitrogen measurements:** C and N contents of 43 samples (ground and sieved <40 µm) were analyzed with an Elementar vario EL cube Analyzer (Elementar, Manchester, UK) at the Friedrich Schiller University Jena. Prior to total organic carbon (TOC) measurements, the samples were pretreated with HCl (10%, 30%) to remove carbonates. Subsequently, the samples were rinsed with ultrapure water to pH neutrality. Of each fraction (untreated and pretreated), up to 30 mg was weighed into tin boats (Elementar, 6 * 6 * 12) and analyzed for total carbon (TC), total nitrogen (TN) and TOC. Total inorganic carbon (TIC) contents were calculated by subtracting TOC from TC. Relative errors estimated for the measurements are based on triplicate analyses (TC ≤ 0.14%, N ≤ 0.44%, TOC ≤ 0.59%; n = 3). The molar TOC/N ratio was calculated as: molar TOC/N = (TOC*12.0107)/(N*14.0067).

**Compound-specific δ^13^C*_n_*_-alkane_ measurements:** δ^13^C*_n_*_-alkane_ analyzes were performed with an isoprime visION isotope ratio mass spectrometer (Elementar, Manchester, UK) coupled via a GC5 pyrolysis–combustion interface (Elementar, Manchester, UK) to an Agilent 7890B gas chromatograph (Agilent, Santa Clara, California, USA) equipped with an Agilent HP5GC column (30m * 320 µm * 0.25 µm film thickness). The GC5 operated in combustion mode (CuO reactor) at 850 °C. Samples were injected in splitless mode and measured in triplicates. *n*-Alkane standards (*n*-C_27_, *n*-C_29_ and *n*-C_33_) with known isotopic composition (Schimmelmann *n*-alkane standards, Indiana, USA) were measured as duplicates after every third triplicate. The standard deviation for the triplicate measurements was < 0.6‰. The compound-specific isotopic composition is given in the delta notation versus VPDB.

**Hemicellulose-derived sugar extraction and compound-specific δ^18^O_sugar_ analyzes:** Hemicellulose-derived sugars of 23 sediment samples (0.1 to 0.2 g) were extracted hydrolytically using 10 ml of 4M trifluoroacetic acid at 105 °C for 4 h^66^. Thereafter, the extracted hemicellulose-derived sugars were vacuum-filtrated using glass fiber filters and humic-like substances and cations were removed using XAD-7 and Dowex 50WX8 columns^67^. The purified extracts were rotary-evaporated and derivatized with methylboronic acid (4 mg in 400 µl pyridine) at 60 °C for 1 h. 3-O-Methyl-Glucose and *α*-Androstane were added as internal standards^67^. Compound-specific δ^18^O_sugar_ measurements were performed on a Trace GC 2000 coupled to a Delta V Advantage IRMS via a pyrolysis reactor (GC IsoLink) and a ConFlo IV interface (all devices from Thermo Fisher Scientific, Bremen, Germany) at the Martin-Luther University Halle-Wittenberg, Halle (Saale). Sample injection occurred in splitless mode and analyzes were performed in triplicate measurements. Derivatized sugar standards with known isotopic composition were measured repeatedly at different concentrations and were used for normalization. δ^18^O values were corrected for drift and amount effects and additionally for the oxygen that became introduced with a carbonyl group during hydrolysis^67^. The standard deviation for the triplicate measurements was < 1.4‰ for fucose. The compound-specific oxygen isotopes are given in the delta notation (δ^18^O_Fucose_) versus the Vienna Standard Mean Ocean Water (VSMOW).

**Supplementary References**

1. Shotten, F. W. An Example of Hard-Water Error in Radiocarbon Dating of Vegetable Matter. *Nature* **240,** 460–461; 10.1038/240460a0 (1972).

2. Steinhilber, F. *et al.* 9,400 years of cosmic radiation and solar activity from ice cores and tree rings. *PNAS* **109,** 5967–5971; 10.1073/pnas.1118965109 (2012).

3. Turney, C. S.M., Coope, G. R., Harkness, D. D., Lowe, J. J. & Walker, M. J.C. Implications for the Dating of Wisconsinan (Weichselian) Late-Glacial Events of Systematic Radiocarbon Age Differences between Terrestrial Plant Macrofossils from a Site in SW Ireland. *Quat. Res.* **53,** 114–121; 10.1006/qres.1999.2087 (2000).

4. Gierga, M. *et al.* Long-stored soil carbon released by prehistoric land use: Evidence from compound-specific radiocarbon analysis on Soppensee lake sediments. *Quat. Sci. Rev.* **144,** 123–131; 10.1016/j.quascirev.2016.05.011 (2016).

5. Heaton, T. J. *et al.* The IntCal20 Approach to Radiocarbon Calibration Curve Construction: A New Methodology Using Bayesian Splines and Errors-in-Variables. *Radiocarbon* **62,** 821–863; 10.1017/RDC.2020.46 (2020).

6. Reimer, P. J. *et al.* The IntCal20 Northern Hemisphere Radiocarbon Age Calibration Curve (0–55 cal kBP). *Radiocarbon* **62,** 725–757; 10.1017/RDC.2020.41 (2020).

7. Wang, X., Xia, D., Wang, T., Xue, X. & Li, J. Dust sources in arid and semiarid China and southern Mongolia: Impacts of geomorphological setting and surface materials. *Geomorphology* **97,** 583–600; 10.1016/j.geomorph.2007.09.006 (2008).

8. Pratte, S., Garneau, M. & Vleeschouwer, F. de. Increased atmospheric dust deposition during the Neoglacial in a boreal peat bog from north-eastern Canada. *Palaeogeogr. Palaeoclimatol. Palaeoecol.* **469,** 34–46; 10.1016/j.palaeo.2016.12.036 (2017).

9. Morrill, C. *et al.* Holocene variations in the Asian monsoon inferred from the geochemistry of lake sediments in central Tibet. *Quat. Res.* **65,** 232–243; 10.1016/j.yqres.2005.02.014 (2006).

10. Margalef, O. *et al.* Environmental processes in Rano Aroi (Easter Island) peat geochemistry forced by climate variability during the last 70kyr. *Palaeogeogr. Palaeoclimatol. Palaeoecol.* **414,** 438–450; 10.1016/j.palaeo.2014.09.025 (2014).

11. Liu, Y. *et al.* Salinity drives archaeal distribution patterns in high altitude lake sediments on the Tibetan Plateau. *FEMS Microbiol. Ecol.* **92**; 10.1093/femsec/fiw033 (2016).

12. Ling, Y. *et al.* High-resolution geochemical record for the last 1100 yr from Lake Toson, northeastern Tibetan Plateau, and its climatic implications. *Quat. Int.* **487,** 61–70; 10.1016/j.quaint.2017.03.067 (2018).

13. Li, M., Wang, J., Zhu, L., Wang, L. & Yi, C. Distribution and formation of monohydrocalcite from surface sediments in Nam Co Lake, Tibet. *Quat. Int.* **263,** 85–92; 10.1016/j.quaint.2012.01.035 (2012).

14. Swainson, I. P. The structure of monohydrocalcite and the phase composition of the beachrock deposits of Lake Butler and Lake Fellmongery, South Australia. *Am. Mineral.* **93,** 1014–1018; 10.2138/am.2008.2825 (2008).

15. Stoffers, P. & Fischbeck, R. Monohydrocalcite in the sediments of Lake Kivu (East Africa). *Sedimentology* **21,** 163–170; 10.1111/j.1365-3091.1974.tb01787.x (1974).

16. Peck, J. A. *et al.* Mid to Late Holocene climate change in north central Mongolia as recorded in the sediments of Lake Telmen. *Palaeogeogr. Palaeoclimatol. Palaeoecol.* **183,** 135–153; 10.1016/S0031-0182(01)00465-5 (2002).

17. Meyers, P. A. Preservation of elemental and isotopic source identification of sedimentary organic matter. *Chem. Geol.* **114,** 289–302; 10.1016/0009-2541(94)90059-0 (1994).

18. Meyers, P. A. Organic geochemical proxies of paleoceanographic, paleolimnologic, and paleoclimatic processes. *Org. Geochem.* **27,** 213–250; 10.1016/S0146-6380(97)00049-1 (1997).

19. Meyers, P. A. & Lallier-Vergés, E. Lacustrine Sedimentary Organic Matter Records of Late Quaternary Paleoclimates. *J. Paleolim.* **21,** 345–372; 10.1023/A:1008073732192 (1999).

20. Struck, J. *et al.* Leaf wax *n*-alkane patterns and compound-specific δ^13^C of plants and topsoils from semi-arid and arid Mongolia. *Biogeosciences* **17,** 567–580; 10.5194/bg-17-567-2020 (2020a).

21. Strobel, P., Struck, J., Zech, R. & Bliedtner, M. The spatial distribution of sedimentary compounds and their environmental implications in surface sediments of Lake Khar Nuur (Mongolian Altai). *Earth Surf. Process. Landforms* **55,** 319; 10.1002/esp.5049 (2021).

22. Ficken, K.J., Li, B., Swain, D.L. & Eglinton, G. An *n*-alkane proxy for the sedimentary input of submerged/floating freshwater aquatic macrophytes. *Org. Geochem.* **31,** 745–749; 10.1016/S0146-6380(00)00081-4 (2000).

23. Strobel, P. *et al.* Precipitation and lake water evaporation recorded by terrestrial and aquatic *n*‐alkane δ^2^H isotopes in Lake Khar Nuur, Mongolia. Geochem. Geophys. Geosyst.; 10.1029/2021GC010234 (2022).

24. Aichner, B. *et al.* Hydroclimate in the Pamirs Was Driven by Changes in Precipitation‐Evaporation Seasonality Since the Last Glacial Period. *Geophys. Res. Lett.* **46,** 13972–13983; 10.1029/2019gl085202 (2019).

25. Sachse, D. *et al.* Molecular Paleohydrology: Interpreting the Hydrogen-Isotopic Composition of Lipid Biomarkers from Photosynthesizing Organisms. *Annu. Rev. Earth Planet. Sci.* **40,** 221–249; 10.1146/annurev-earth-042711-105535 (2012).

26. Rach, O., Brauer, A., Wilkes, H. & Sachse, D. Delayed hydrological response to Greenland cooling at the onset of the Younger Dryas in western Europe. *Nat. Geosci.* **7,** 109–112; 10.1038/ngeo2053 (2014).

27. Strobel, P. *et al.* The potential of δ^2^H*_n_*_-alkanes_ and δ^18^O_sugar_ for paleoclimate reconstruction - A regional calibration study for South Africa. *Sci. Total Environ.* **716,** 137045; 10.1016/j.scitotenv.2020.137045 (2020).

28. Mügler, I. *et al.* Effect of lake evaporation on δD values of lacustrine n-alkanes: A comparison of Nam Co (Tibetan Plateau) and Holzmaar (Germany). *Org. Geochem.* **39,** 711–729; 10.1016/j.orggeochem.2008.02.008 (2008).

29. Struck, J. *et al.* Leaf Waxes and Hemicelluloses in Topsoils Reflect the δ^2^H and δ^18^O Isotopic Composition of Precipitation in Mongolia. *Front. Earth Sci.* **8,** 619; 10.3389/feart.2020.00343 (2020b).

30. Dykoski, C. A. *et al.* A high-resolution, absolute-dated Holocene and deglacial Asian monsoon record from Dongge Cave, China. *Earth Planet. Sci. Lett.* **233,** 71–86; 10.1016/j.epsl.2005.01.036 (2005).

31. Diefendorf, A. F. & Freimuth, E. J. Extracting the most from terrestrial plant-derived *n*-alkyl lipids and their carbon isotopes from the sedimentary record: A review. *Org. Geochem.* **103,** 1–21; 10.1016/j.orggeochem.2016.10.016 (2017).

32. Aichner, B., Feakins, S. J., Lee, J. E., Herzschuh, U. & Liu, X. High-resolution leaf wax carbon and hydrogen isotopic record of the late Holocene paleoclimate in arid Central Asia. *Clim. Past* **11,** 619–633; 10.5194/cp-11-619-2015 (2015).

33. Enders, S. K. *et al.* Compound-specific stable isotopes of organic compounds from lake sediments track recent environmental changes in an alpine ecosystem, Rocky Mountain National Park, Colorado. *Limnol. Oceanogr.* **53,** 1468–1478; 10.4319/lo.2008.53.4.1468 (2008).

34. Horton, T. W., Defliese, W. F., Tripati, A. K. & Oze, C. Evaporation induced ^18^O and ^13^C enrichment in lake systems: A global perspective on hydrologic balance effects. *Quat. Sci. Rev.* **131,** 365–379; 10.1016/j.quascirev.2015.06.030 (2016).

35. Hepp, J. *et al.* A sugar biomarker proxy for assessing terrestrial versus aquatic sedimentary input. *Org. Geochem.* **98,** 98–104; 10.1016/j.orggeochem.2016.05.012 (2016).

36. Lan, J. *et al.* Late Holocene hydroclimatic variation in central Asia and its response to mid-latitude Westerlies and solar irradiance. *Quat. Sci. Rev.* **238,** 106330; 10.1016/j.quascirev.2020.106330 (2020).

37. Wünnemann, B. *et al.* A 14 ka high-resolution δ^18^O lake record reveals a paradigm shift for the process-based reconstruction of hydroclimate on the northern Tibetan Plateau. *Quat. Sci. Rev.* **200,** 65–84; 10.1016/j.quascirev.2018.09.040 (2018).

38. Xu, H., Ai, L., Tan, L. & An, Z. Stable isotopes in bulk carbonates and organic matter in recent sediments of Lake Qinghai and their climatic implications. *Chem. Geol.* **235,** 262–275; 10.1016/j.chemgeo.2006.07.005 (2006).

39. Lei, Y. *et al.* Characteristics of δ^13^C_DIC_ in lakes on the Tibetan Plateau and its implications for the carbon cycle. *Hydrol. Process.* **26,** 535–543; 10.1002/hyp.8152 (2012).

40. Leng, M. J. & Marshall, J. D. Palaeoclimate interpretation of stable isotope data from lake sediment archives. *Quat. Sci. Rev.* **23,** 811–831; 10.1016/j.quascirev.2003.06.012 (2004).

41. van der Meeren, T., Almendinger, J. E., Ito, E. & Martens, K. The ecology of ostracodes (Ostracoda, Crustacea) in western Mongolia. *Hydrobiologia* **641,** 253–273; 10.1007/s10750-010-0089-y (2010).

42. Chikaraishi, Y. & Naraoka, H. Compound-specific δD–δ^13^C analyses of *n*-alkanes extracted from terrestrial and aquatic plants. *Phytochemistry* **63,** 361–371; 10.1016/S0031-9422(02)00749-5 (2003).

43. Sachse, D., Radke, J. & Gleixner, G. Hydrogen isotope ratios of recent lacustrine sedimentary *n*-alkanes record modern climate variability. *Geochim. Cosmochim. Acta* **68,** 4877–4889; 10.1016/j.gca.2004.06.004 (2004).

44. Sessions, A. L., Burgoyne, T. W., Schimmelmann, A. & Hayes, J. M. Fractionation of hydrogen isotopes in lipid biosynthesis. *Org. Geochem.* **30,** 1193–1200; 10.1016/S0146-6380(99)00094-7 (1999).

45. Sessions, A. L. Seasonal changes in D/H fractionation accompanying lipid biosynthesis in Spartina alterniflora. *Geochim. Cosmochim. Acta* **70,** 2153–2162; 10.1016/j.gca.2006.02.003 (2006).

46. Guenther, F. *et al.* A synthesis of hydrogen isotope variability and its hydrological significance at the Qinghai–Tibetan Plateau. *Quat. Int.* **313-314,** 3–16; 10.1016/j.quaint.2013.07.013 (2013).

47. Aichner, B., Hilt, S., Périllon, C., Gillefalk, M. & Sachse, D. Biosynthetic hydrogen isotopic fractionation factors during lipid synthesis in submerged aquatic macrophytes: Effect of groundwater discharge and salinity. *Org. Geochem.* **113,** 10–16; 10.1016/j.orggeochem.2017.07.021 (2017).

48. Soninkhishig, N., Edlund, M. B. & Peck, J. A. Diatom-Based Paleoenvironmental Reconstruction of Lake Telmen for the Last 6230 Years. *Mong. J. Biol. Sci.* **1,** 55–68; 10.22353/mjbs.2003.01.06 (2003).

49. Minderlein, S. & Menzel, L. Evapotranspiration and energy balance dynamics of a semi-arid mountainous steppe and shrubland site in Northern Mongolia. *Environ. Earth Sci.* **73,** 593–609; 10.1007/s12665-014-3335-1 (2015).

50. Nandintsetseg, B. & Shinoda, M. Multi-Decadal Soil Moisture Trends in Mongolia and Their Relationships to Precipitation and Evapotranspiration. *Arid Land Res. Manage.* **28,** 247–260; 10.1080/15324982.2013.861882 (2014).

51. Yang, X., Rost, K. T., Lehmkuhl, F., Zhenda, Z. & Dodson, J. The evolution of dry lands in northern China and in the Republic of Mongolia since the Last Glacial Maximum. *Quat. Int.* **118-119,** 69–85; 10.1016/S1040-6182(03)00131-9 (2004).

52. Dugerdil, L. *et al.* Late Holocene Mongolian climate and environment reconstructions from brGDGTs, NPPs and pollen transfer functions for Lake Ayrag: Paleoclimate implications for Arid Central Asia. *Quat. Sci. Rev.* **273,** 107235; 10.1016/j.quascirev.2021.107235 (2021).

53. Bliedtner, M. *et al.* Late Holocene Climate Changes in the Altai Region Based on a First High‐Resolution Biomarker Isotope Record From Lake Khar Nuur. *Geophys. Res. Lett.* **48**; 10.1029/2021GL094299 (2021).

54. Büntgen, U. *et al.* Cooling and societal change during the Late Antique Little Ice Age from 536 to around 660 AD. *Nat. Geosci..* **9,** 231–236; 10.1038/NGEO2652 (2016).

55. Klinge, M. & Sauer, D. Spatial pattern of Late Glacial and Holocene climatic and environmental development in Western Mongolia - A critical review and synthesis. *Quat. Sci. Rev.* **210,** 26–50; 10.1016/j.quascirev.2019.02.020 (2019).

56. Yang, Y., Ran, M. & Sun, A. Pollen‐recorded bioclimatic variations of the last ~2000 years retrieved from Bayan Nuur in the western Mongolian Plateau. *Boreas* **49,** 350–362; 10.1111/bor.12423 (2020).

57. Klinge, M., Lehmkuhl, F., Schulte, P., Hülle, D. & Nottebaum, V. Implications of (reworked) aeolian sediments and paleosols for Holocene environmental change in Western Mongolia. *Geomorphology* **292,** 59–71; 10.1016/j.geomorph.2017.04.027 (2017).

58. Lehmkuhl, F., Grunert, J., Hülle, D., Batkhishig, O. & Stauch, G. Paleolakes in the Gobi region of southern Mongolia. *Quat. Sci. Rev.* **179,** 1–23; 10.1016/j.quascirev.2017.10.035 (2018).

59. Rudaya, N. *et al.* Holocene environments and climate in the Mongolian Altai reconstructed from the Hoton-Nur pollen and diatom records: a step towards better understanding climate dynamics in Central Asia. *Quat. Sci. Rev.* **28,** 540–554; 10.1016/j.quascirev.2008.10.013 (2009).

60. Lan, J. *et al.* Dramatic weakening of the East Asian summer monsoon in northern China during the transition from the Medieval Warm Period to the Little Ice Age. *Geology* **48,** 307–312; 10.1130/G46811.1 (2020).

61. Pederson, N., Hessl, A. E., Baatarbileg, N., Anchukaitis, K. J. & Di Cosmo, N. Pluvials, droughts, the Mongol Empire, and modern Mongolia. *PNAS* **111,** 4375–4379; 10.1073/pnas.1318677111 (2014).

62. Unkelbach, J., Kashima, K., Punsalpaamuu, G., Shumilovskikh, L. & Behling, H. Decadal high-resolution multi-proxy analysis to reconstruct natural and human-induced environmental changes over the last 1350 cal. yr BP in the Altai Tavan Bogd National Park, western Mongolia. *The Holocene* **30,** 1016–1028; 10.1177/0959683620908662 (2020).

63. Szidat, S. *et al.* 14 C Analysis and Sample Preparation at the New Bern Laboratory for the Analysis of Radiocarbon with AMS (LARA). *Radiocarbon* **56,** 561–566; 10.2458/56.17457 (2014).

64. Salazar, G., Zhang, Y. L., Agrios, K. & Szidat, S. Development of a method for fast and automatic radiocarbon measurement of aerosol samples by online coupling of an elemental analyzer with a MICADAS AMS. *Nucl. Instrum. Methods Phys. Res., Sect. B* **361,** 163–167; 10.1016/j.nimb.2015.03.051 (2015).

65. Blaauw, M. & Christen, J. A. Flexible paleoclimate age-depth models using an autoregressive gamma process. *Bayesian Anal.* **6,** 457–474; 10.1214/11-BA618 (2011).

66. Amelung, W., Cheshire, M. V. & Guggenberger, G. Determination of neutral and acidic sugars in soil by capillary gas-liquid chromatography after trifluoroacetic acid hydrolysis. *Soil Biol. Biochem.* **28,** 1631–1639; 10.1016/S0038-0717(96)00248-9.

67. Zech, M. & Glaser, B. Compound-specific δ^18^O analyses of neutral sugars in soils using gas chromatography-pyrolysis-isotope ratio mass spectrometry: problems, possible solutions and a first application. *Rapid Commun. Mass Spectrom.* **23,** 3522–3532; 10.1002/rcm.4278 (2009).
